# A cancer-associated Epstein-Barr virus BZLF1 promoter variant enhances lytic infection

**DOI:** 10.1371/journal.ppat.1007179

**Published:** 2018-07-27

**Authors:** Jillian A. Bristol, Reza Djavadian, Emily R. Albright, Carrie B. Coleman, Makoto Ohashi, Mitchell Hayes, James C. Romero-Masters, Elizabeth A. Barlow, Paul J. Farrell, Rosemary Rochford, Robert F. Kalejta, Eric C. Johannsen, Shannon C. Kenney

**Affiliations:** 1 Department of Oncology in Wisconsin Institutes for Medical Research, McArdle Laboratory for Cancer Research, University of Wisconsin School of Medicine and Public Health, Madison, Wisconsin, United States of America; 2 Department of Molecular Virology, University of Wisconsin School of Medicine and Public Health, Madison, Wisconsin, United States of America; 3 Department of Immunology & Microbiology, University of Colorado School of Medicine, Denver, Colorado, United States of America; 4 Department of Pathology and Laboratory Medicine, McArdle Laboratory for Cancer Research, University of Wisconsin School of Medicine and Public Health, Madison, Wisconsin, United States of America; 5 Molecular Virology, Department of Medicine, Imperial College London, London, United Kingdom; 6 Department of Immunology and Microbiology, University of Colorado, Aurora, Colorado United States of America; 7 Department of Medicine, McArdle Laboratory for Cancer Research, University of Wisconsin School of Medicine and Public Health, Madison, Wisconsin, United States of America; Tulane Health Sciences Center, UNITED STATES

## Abstract

Latent Epstein-Barr virus (EBV) infection contributes to both B-cell and epithelial-cell malignancies. However, whether lytic EBV infection also contributes to tumors is unclear, although the association between malaria infection and Burkitt lymphomas (BLs) may involve excessive lytic EBV replication. A particular variant of the viral promoter (Zp) that controls lytic EBV reactivation is over-represented, relative to its frequency in non-malignant tissue, in EBV-positive nasopharyngeal carcinomas and AIDS-related lymphomas. To date, no functional differences between the prototype Zp (Zp-P) and the cancer-associated variant (Zp-V3) have been identified. Here we show that a single nucleotide difference between the Zp-V3 and Zp-P promoters creates a binding site for the cellular transcription factor, NFATc1, in the Zp-V3 (but not Zp-P) variant, and greatly enhances Zp activity and lytic viral reactivation in response to NFATc1-inducing stimuli such as B-cell receptor activation and ionomycin. Furthermore, we demonstrate that restoring this NFATc1-motif to the Zp-P variant in the context of the intact EBV B95.8 strain genome greatly enhances lytic viral reactivation in response to the NFATc1-activating agent, ionomycin, and this effect is blocked by the NFAT inhibitory agent, cyclosporine, as well as NFATc1 siRNA. We also show that the Zp-V3 variant is over-represented in EBV-positive BLs and gastric cancers, and in EBV-transformed B-cell lines derived from EBV-infected breast milk of Kenyan mothers that had malaria during pregnancy. These results demonstrate that the Zp-V3 enhances EBV lytic reactivation to physiologically-relevant stimuli, and suggest that increased lytic infection may contribute to the increased prevalence of this variant in EBV-associated malignancies.

## Author summary

Whether excessive lytic EBV infection increases the risk of EBV-induced cancers is not clear. A particular variant (Zp-V3) of the viral promoter driving expression of the EBV immediate-early BZLF1 (Z) protein that mediates lytic viral reactivation has been reported to be over-represented (relative to the prototype Zp-P form of the promoter) in certain EBV-positive malignancies, but no functional difference between the two promoter variants has been reported. Here we show that the malignancy-associated Zp-V3 variant (but not the Zp-P variant) contains a binding site for the cellular NFATc1 (nuclear factor of activated T cells c1) transcription factor that allows it to be activated by NFATc1-inducing stimuli such as B-cell receptor stimulation. Furthermore, we demonstrate that restoring this NFATc1-motif to the Zp-P variant in the context of the intact EBV genome greatly enhances lytic viral reactivation in response to the NFATc1-inducing stimuli. We also find that the Zp-V3 variant is over-represented in EBV-positive Burkitt lymphomas and gastric carcinomas, and in lymphoblastoid cell lines transformed by EBV-infected breast milk of Kenyan mothers that had malaria during pregnancy. These findings suggest that the Zp-V3 version of the EBV BZLF1 promoter increases the likelihood of EBV-induced malignancies by increasing lytic EBV infection.

## Introduction

Epstein-Barr virus (EBV) causes infectious mononucleosis and is associated with a variety of different types of human malignancies, including B-cell lymphomas and nasopharyngeal carcinoma. EBV infects approximately 90% of the world’s population, and like all herpesviruses, EBV persists in the host for life. EBV establishes long-term viral latency in the memory B cell compartment of humans [1–3], whereas lytic EBV infection occurs in antigen-stimulated B cells, plasma cells and oropharyngeal epithelial cells [4–6]. While the initial viral infection is often associated with the clinical symptoms of infectious mononucleosis, EBV does not usually cause any subsequent illness in immune competent hosts, although infectious viral particles continue to be periodically shed in the saliva.

Nevertheless, EBV infection sometimes leads to the development of EBV-positive tumors, particularly in immunosuppressed individuals. EBV-induced tumors are thought to be primarily due to the latent form of viral infection. Latent EBV infection transforms primary human B cells *in vitro* into immortalized lymphoblastoid cell lines (LCLs) that induce lymphomas when injected into immunodeficient mice. Furthermore, the major EBV-encoded oncoproteins are expressed during latent EBV infection, and EBV-positive tumors in humans are composed largely of latently infected cells.

Given the essential roles of the viral latency proteins in EBV-positive tumors, relatively little attention has been paid to the potential role(s) of lytic EBV infection in promoting EBV-induced tumors. Although widespread fully lytic EBV infection of tumor cells would likely be incompatible with tumor growth, increasing evidence suggests that lytic viral infection contributes to the formation of tumors caused by the related human gamma herpesvirus, KSHV (Kaposi’s sarcoma associated herpesvirus) [7]. Excessive lytic EBV infection in humans could potentially increase the likelihood of EBV-positive tumors by increasing the total number of virally infected cells (including the number of latently infected cells), and/or by inducing paracrine effects that help support the growth and/or viability of latently infected tumor cells. For example, B cells with lytic EBV infection have enhanced secretion of the B cell growth factor, IL-6 [8], the angiogenesis factor, VEGF [9], and the immunosuppressive factors, viral and cellular IL-10 [10,11].

Consistent with a role for lytic EBV infection in promoting the development of EBV-associated tumors, increased lytic infection preceding tumor development seems to occur in a number of EBV-positive human cancers. Patients who develop EBV-positive nasopharyngeal carcinoma (NPC) almost universally have extremely high levels of antibodies directed against lytic EBV proteins even before their cancers are clinically symptomatic, and monitoring lytic EBV antibody levels is useful for detecting NPC at early stages [12]. Immunosuppressed organ transplant recipients, who are highly prone to developing EBV-induced lymphoproliferative disease, have a high level of lytic, as well as latent, EBV infection [13]. Malaria infection, which is thought to promote the development of EBV-positive Burkitt lymphoma (BL), greatly increases the amount of lytic EBV infection in children [14–16]. Furthermore, higher levels of infectious EBV are present in the breast milk of malaria-infected, versus malaria-uninfected, Kenyan women [17], and Kenyan infants more frequently become EBV-infected at very young ages (less than 6 months) when residing in areas with a high prevalence of malaria [18].

The latent-to-lytic switch in EBV-infected cells is mediated by the EBV immediate-early BZLF1 gene product (Z). Z is a transcription factor that binds to, and transcriptionally activates, lytic viral gene promoters, resulting in the lytic form of viral DNA replication and assembly of infectious viral particles [19]. Regulation of the Z promoter (Zp) by cellular transcription factors determines whether EBV infection is latent or lytic. B-cell receptor (BCR) stimulation potently induces lytic EBV gene expression in certain Burkitt lymphoma cell lines *in vitro*, and BCR activation in response to antigen stimulation of EBV-infected B cells is thought to be a biologically important mechanism by which the EBV life cycle is regulated in humans [19]. In addition to its immunosuppressive effect [20], malaria is thought to increase the amount of lytic EBV infection by inducing polyclonal B cell stimulation [16,21]. However, the precise cellular transcription factors that link the BCR signal to EBV lytic reactivation are only partially understood, and EBV-infected cell lines differ substantially in their ability to reactivate in response to BCR stimulation *in vitro*.

A particular promoter variant of the EBV Z promoter (Zp-V3) has been reported in two different studies to be over-represented in both EBV-infected NPCs [22] and EBV-infected AIDS-related lymphomas [23] in comparison to its frequency in EBV-infected non-malignant tissues obtained from patients in the same geographic regions. However, whether this promoter variant affects BZLF1 transcription or lytic EBV reactivation is not known. Here, we have examined the functional consequences of the malignancy-associated Zp-V3 variant. We show that in comparison to the prototype Zp variant (Zp-P), the Zp-V3 variant responds much more strongly to BCR-ligation and ionomycin stimulation in B cells. Furthermore, we demonstrate that this difference results from a single nucleotide difference in the two promoter variants (which creates an NFATc1 binding motif in the Zp-V3 form of the promoter), and is sufficient to confer greatly enhanced lytic viral protein expression in EBV-infected B cells. Importantly, we find that the Zp-V3 variant is highly over-represented in a set of EBV-transformed lymphoblastoid cell lines (LCLs) that were derived from EBV present in breast milk of Kenyan mothers that had malaria during pregnancy, versus a set of spontaneous LCLs that were derived from the blood of healthy Kenyan individuals living in malaria-high regions. In addition, we show that the Zp-V3 variant is also over-represented in both EBV-positive Burkitt lymphomas, and EBV-positive gastric carcinomas, relative to its frequency in healthy control patients. These findings suggest that the Zp-V3 version of the EBV BZLF1 promoter increases the likelihood of EBV-induced malignancies by increasing the level of lytic EBV infection.

## Results

### The Zp-V3 promoter variant has enhanced activation in response to B-cell receptor (BCR) stimulation

**Fig 1:**
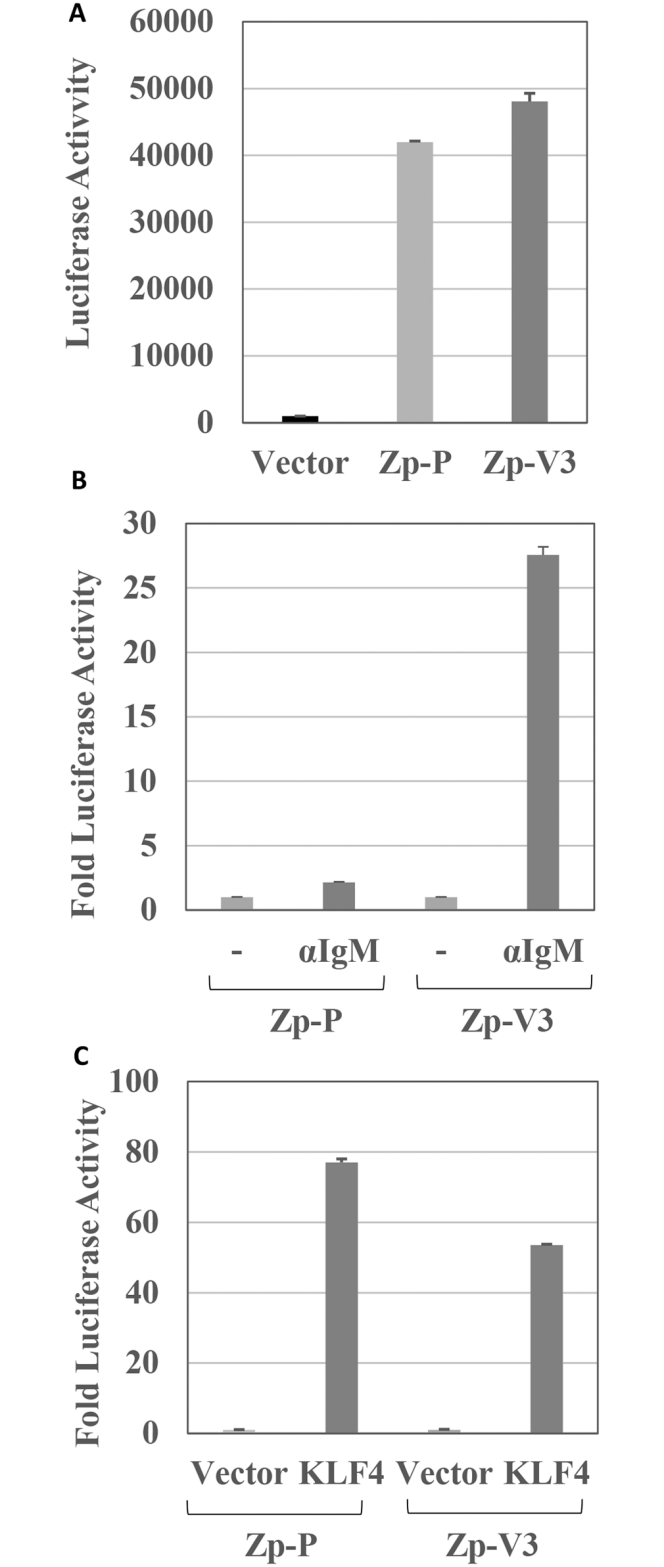
Zp-V3 is much more responsive to BCR crosslinking than Zp-P. **(A)** EBV-negative BJAB cells were transfected with a promoterless luciferase construct, or luciferase constructs driven by the Zp-P or Zp-V3 promoters, and luciferase activity was measured after 24 hours. The amount of luciferase activity for each condition (average and the standard deviation, SD) is shown. **(B)** BJAB cells were transfected with luciferase constructs driven by the Zp-P or Zp-V3 promoters, and then treated with or without anti-human IgM (10 mg/mL) 24 hours later to activate the B cell receptor (BCR). Luciferase activity was measured at 48 hours after transfection and results were normalized to that each promoter’s untreated condition (set as 1). The fold increase in luciferase activity is shown for each condition (average and SD). **(C)** EBV-negative NOKs epithelial cells were transfected with the Zp-P or Zp-V3 luciferase vectors, and a KLF4 expression vector or vector control as indicated, and luciferase activity was measured 24 hours post-transfection. The amount of luciferase activity for each condition (average and SD) is shown and results were normalized to that of each promoter’s untreated condition (set as 1).

To determine if the Zp-P and Zp-V3 variants of the EBV Z promoter have different activities in a B-cell environment, we inserted the two promoter variants upstream of the luciferase gene in the pCpGL luciferase vector and performed transient reporter gene assays. Although the two promoter variants had similar constitutive activity in BJAB cells (derived from an EBV-negative B-cell lymphoma) (Fig 1A), the Zp-V3 variant was much more efficiently activated when cells were treated with an anti-IgM antibody to stimulate the BCR (Fig 1B). In contrast to the effect of BCR stimulation, the two promoter variants responded similarly to the KLF4 transcription factor (Fig 1C), which binds to and activates the Z promoter [24,25]. These results suggest that the Zp-V3 variant of the Z promoter is more responsive to BCR stimulation than the Zp-P variant.

### Residue -141 in the Zp-V3 promoter variant is required for efficient BCR stimulation

**Fig 2:**
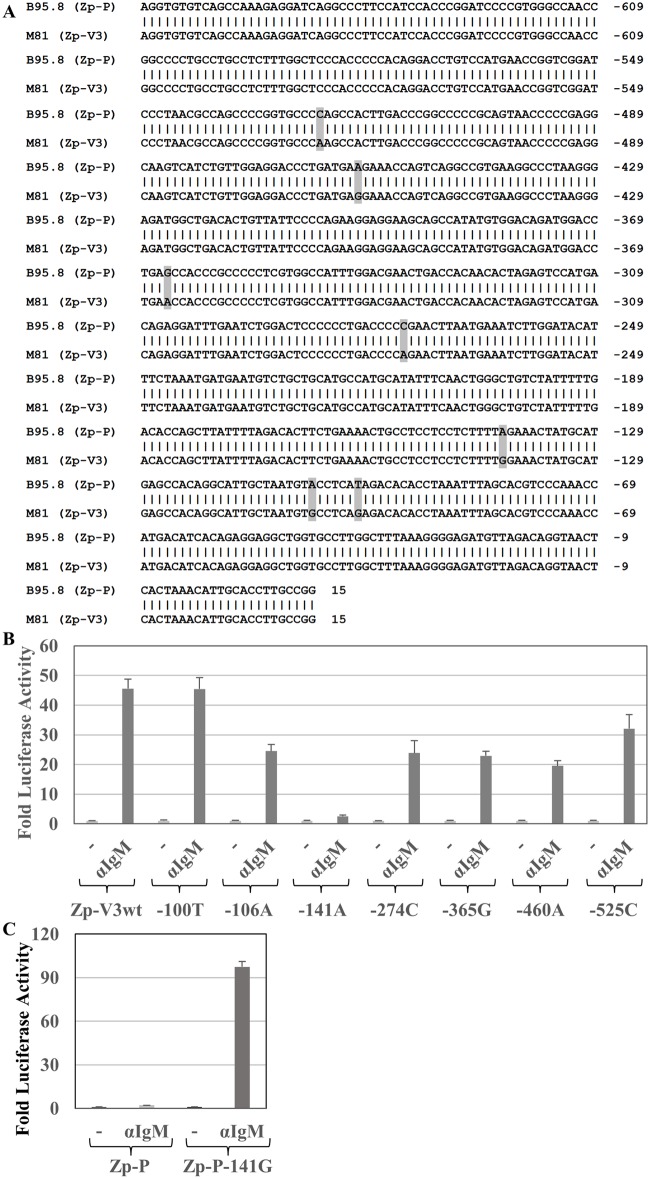
Residue -141 in the Zp-V3 promoter is critical for response to BCR crosslinking. **(A)** The Z promoter sequences in the B95.8 (top) and M81 (bottom) EBV strains are shown. B95.8 is the prototype (Zp-P) while M81 has the 3 polymorphisms at -100, -106, and -141 that define Variant 3 (Zp-V3). There are 4 additional basepair differences between the two Zp sequences, located at -274, -365, -460, and -525 (relative to the transcriptional start site), highlighted in gray. **(B)** EBV-negative BJAB cells were transfected with wildtype or mutant Zp-V3 luciferase constructs (named to reflect the promoter basepair altered in Zp-V3 relative to the Zp start site) and treated with or without anti-IgM. The luciferase activity for each construct is shown and results were normalized to that of each promoter’s untreated condition (set as 1). **(C)** BJAB cells were transfected with either the wildtype Zp-P luciferase construct or a mutant Zp-P construct in which the A nucleotide located at -141 is switched to the G nucleotide found in the Zp-V3 sequence. Cells were treated with or without anti-IgM 24 hours after transfection. Luciferase activity was measured at 48 hours after transfection and results were normalized to that of each promoter’s untreated condition (set as 1). The fold increase in luciferase activity is shown for each condition (average and SD).

The promoter sequences located between -668 and +15 (relative to the transcriptional start site) of the Zp-P (from B95.8 strain EBV) and Zp-V3 (from M81 strain EBV) variants differ by only seven nucleotides (Fig 2A). To examine how these differences contribute to the enhanced responsiveness of the Zp-V3 variant to BCR stimulation, we individually switched each of the variant nucleotides in the Zp-V3 luciferase construct to the nucleotides present in the Zp-P promoter. Altering the variant residues located at -100, -106, -274, -365, -460, and -525 (relative to the transcriptional start site) had relatively little effect on the response of the Zp-V3 promoter to anti-IgM treatment in BJAB cells (Fig 2B). However, switching the nucleotide located at -141 from a G to an A almost completely abolished the ability of the Zp-V3 variant to be activated by BCR stimulation, reducing it to the level seen with Zp-P. Thus, a G nucleotide at position -141 in the Zp promoter is required for efficient BCR activation.

To determine if a G nucleotide at -141 is sufficient to increase the BCR responsiveness of the Zp-P version of the Z promoter, we switched the -141 nucleotide from an A to G in the Zp-P luciferase construct. As shown in Fig 2C, this single basepair change conferred strong responsiveness to BCR stimulation. These results suggest that a single nucleotide difference in the two Z promoter variants is largely responsible for the different responses of Zp-V3 and Zp-P to BCR stimulation.

### The NFATc1 transcription factor binds to the -141 region of Zp-V3, but not Zp-P

**Fig 3:**
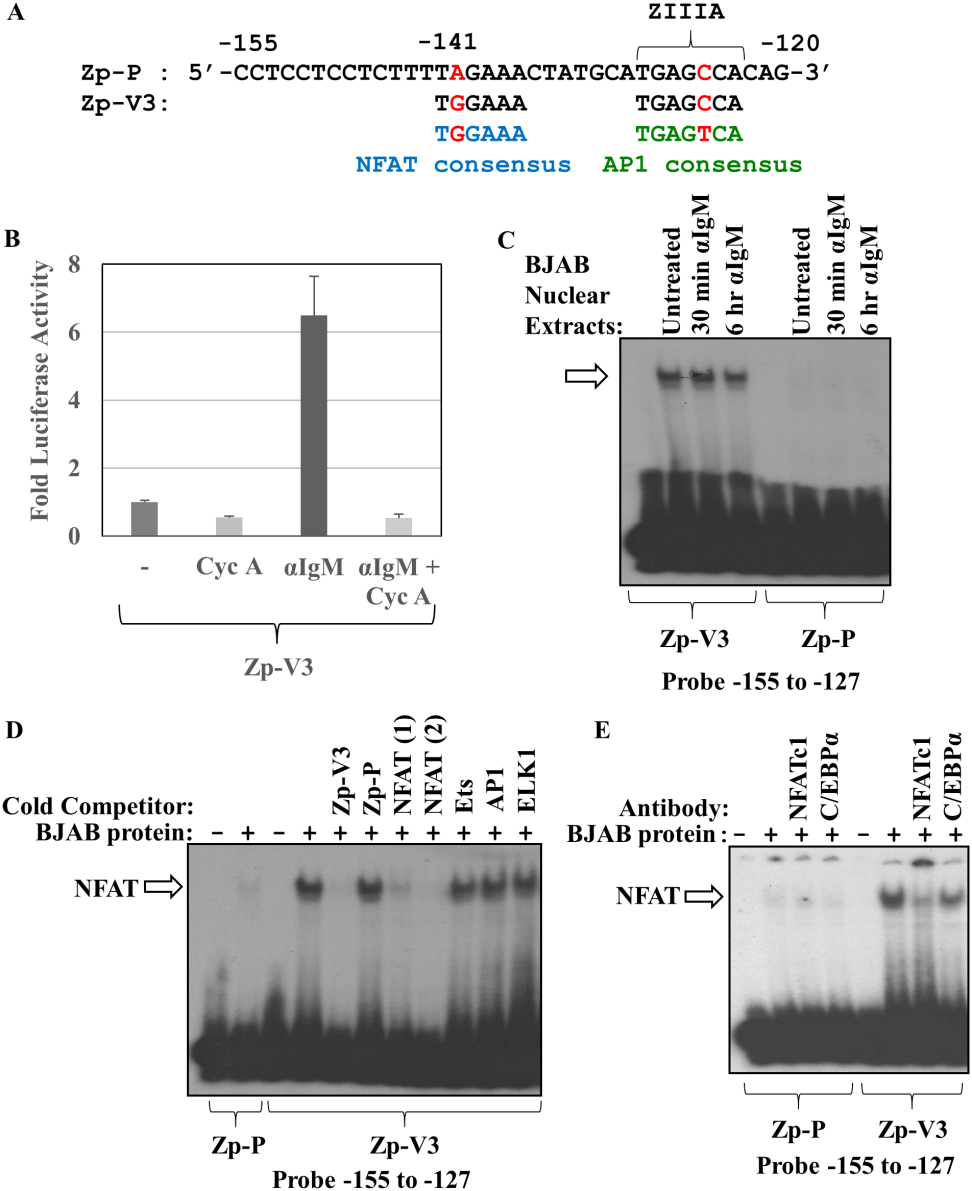
Zp-V3 (but not Zp-P) binds NFATc1. **(A)** EMSA oligonucleotides were designed to encompass the regions of Zp-V3 and Zp-P from -155 to -127, or from -155 to -120 and radiolabeled with ^32^P. The potential NFAT (in Zp-V3, not Zp-P) and AP1 binding sites (in both variants) are indicated. **(B)** EBV-negative BJAB cells were transfected with the Zp-V3 luciferase vector, and then treated 24 hours later with or without anti-IgM, in the presence or absence of cyclosporine A (1μM) as indicated. Luciferase activity was measured 24 hours after anti-IgM treatment and results were normalized to that of the Zp-V3 untreated condition. (set as 1). The fold increase in luciferase activity is shown for each condition (average and SD). **(C)** Nuclear extracts prepared from BJAB cells, treated with or without anti-IgM for 30 minutes or 6 hours, were incubated with the radiolabeled Zp-P or Zp-V3 (-155 to -127) probes in an EMSA. A protein that binds only to the Zp-V3 probe is indicated by an arrow. **(D)** EMSA was performed using radiolabeled Zp-P and Zp-V3 probes (-155 to -127) and untreated nuclear BJAB extract. Cold competitor DNA containing either the Zp-P or Zp-V3 oligonucleotides, or the consensus binding sites for the transcription factors indicated, was added in some conditions. An arrow depicts bands representing NFAT binding. **(E)** EMSA was performed using radiolabeled Zp-P and Zp-V3 probes (-155 to -127) and untreated nuclear BJAB extract. In some conditions, antibodies against NFATc1 or C/EBPα were added to the nuclear extract (prior to the addition of the labeled probe) as shown. An arrow depicts bands representing NFAT binding.

Comparison of the Zp-V3 versus Zp-P sequences surrounding the -141 Zp nucleotide revealed that the Zp-V3 promoter has a consensus NFAT (Nuclear Factor of Activated T cells) motif (TGGAAA) [26] that is not present in the Zp-P version of the promoter (Fig 3A). The NFAT family consists of five cellular transcription factors; NFATc1, expressed in lymphoid tissue, is translocated to the nucleus following T cell receptor and BCR activation [27–30], and is constitutively activated in some B cell lymphomas, including the BJAB cell line [31–33]. Since BCR signaling is known to translocate and activate cellular NFAT transcription factors [30,34], we used a luciferase assay to determine whether pretreating BJAB cells with cyclosporine (an NFAT inhibitor [27]) abolished the ability of BCR stimulation to activate the Zp-V3 promoter. As shown in Fig 3B, cyclosporine treatment prevented anti-IgM activation of Zp-V3, suggesting that the BCR stimulatory effect may be mediated through a NFAT family member. Of note, NFAT activation of promoters commonly requires cooperative binding of NFAT with other transcription factors (in particular, AP1 and Ets family members) [35–38], and the potential NFAT site on the Zp-V3 variant is adjacent to an AP1-like motif (TGAGCCA) known as ZIIIA that has previously been shown to be required for BCR activation of Zp [39,40].

To determine if NFATc1 can bind to the Zp-V3, but not Zp-P, version of the Z promoter, we performed EMSAs using labeled oligonucleotide probes containing the -155 to -127 sequences of either Zp variant, and nuclear extracts harvested from untreated or anti-IgM treated BJAB cells. These probes contain the potential NFAT site but not the adjacent potential AP1 motif. A protein that binds to the Zp-V3, but not Zp-P, version of the probes was observed in the presence and absence of anti-IgM treatment (Fig 3C). Furthermore, two different unlabeled oligonucleotides containing different known binding sequences for NFAT competed for binding with this protein, while oligonucleotides containing Ets, AP1, and ELK1 consensus sites did not (Fig 3D). In addition, pre-incubating the nuclear extract with an antibody against NFATc1 blocked most of the binding to the Zp-V3 probe, while an antibody against another transcription factor, C/EBPα, had little effect (Fig 3E). These results confirm that NFATc1 can bind to the Zp-V3 but not Zp-P form of Zp.

### NFATc1 recruits AP1 to the -141 region of Zp-V3

**Fig 4:**
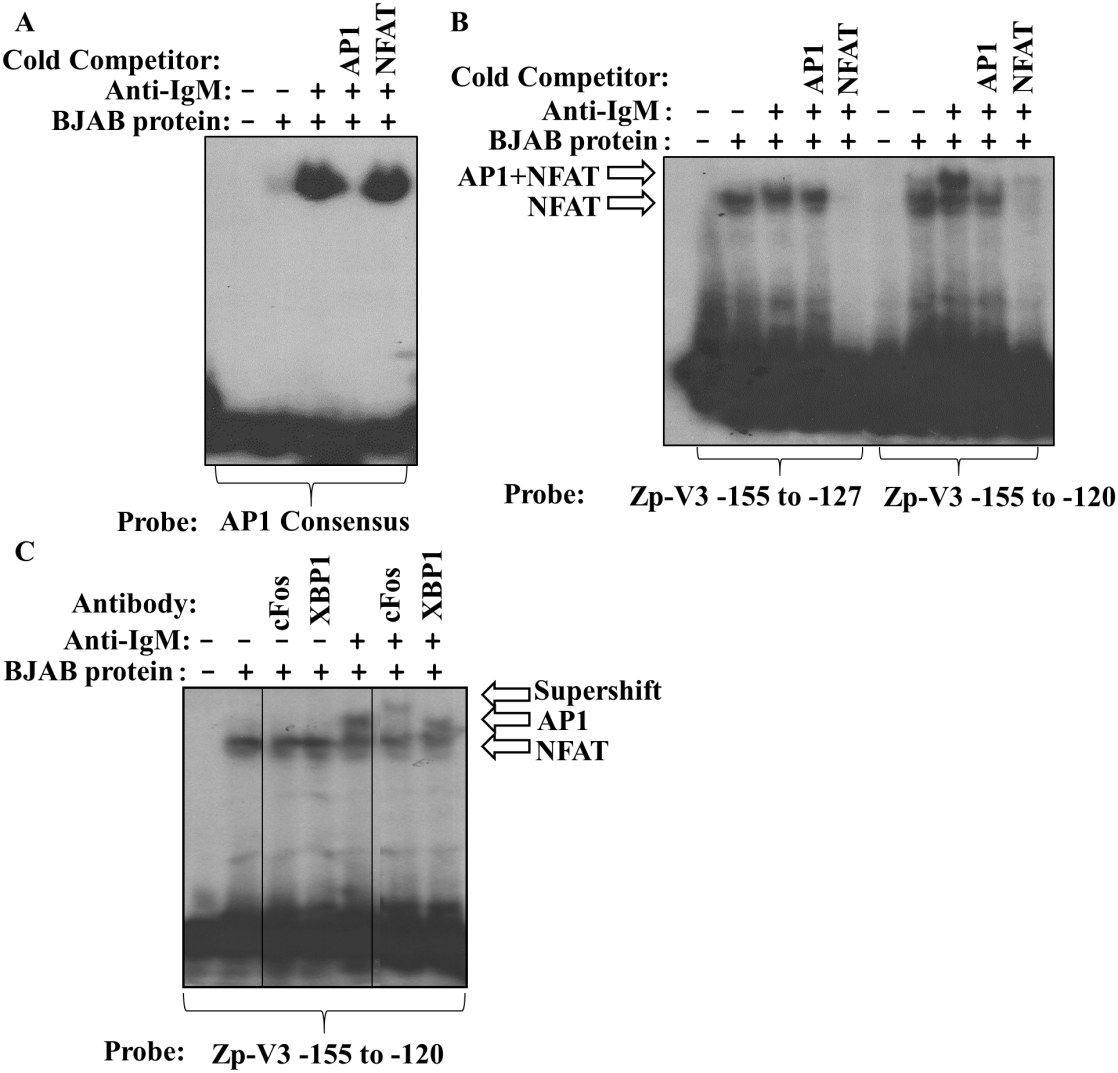
The longer Zp-V3 probe contains a cooperative NFAT/AP1 binding motif. EBV-negative BJAB nuclear extracts, with or without exposure to 6 hours of anti-IgM, were incubated with radiolabeled probes in EMSA assays. **(A)** A radiolabeled AP1 consensus probe was incubated with nuclear extract obtained from untreated or anti-IgM treated BJAB cells and EMSA performed. Cold competitor DNA containing consensus binding sites for the AP1 or NFAT transcription factors was added in some conditions. **(B)** Radiolabeled probes containing the Zp-V3 sequences (either from -155 to -127, or from -155 to -120 as indicated) were incubated with nuclear extract obtained from untreated or anti-IgM treated BJAB cells and EMSA performed. Cold competitor DNA containing consensus binding sites for the AP1 or NFAT transcription factors was added in some conditions. Arrows depict bands representing NFAT binding alone and NFAT plus AP1 binding. **(C)** A radiolabeled probe containing the Zp-V3 sequence from -155 to -120 was incubated with nuclear extract obtained from untreated or anti-IgM treated BJAB cells and EMSA performed. In some conditions, antibodies against cFos or XBP1 were added to the nuclear extract (prior to the addition of the labeled probe) as shown. Arrows depict bands representing NFAT binding alone, NFAT plus AP1 binding, and the NFAT plus AP1 band that is supershifted by the cFos antibody.

Since NFATc1 is constitutively expressed in BJAB nuclear extracts, and its binding to Zp-V3 is not increased by anti-IgM treatment of cells, we next asked if BCR stimulation of BJAB cells enhances AP1 binding to the adjacent AP1-like (“ZIIIA”) motif, and if this effect is NFATc1-dependent. To confirm that crosslinking of the BCR (which is known to induce expression of AP1 family members [24,25]) results in enhanced AP1 activity in BJAB cells, EMSAs were performed using a labeled consensus AP1 probe and nuclear extracts harvested from untreated or anti-IgM treated cells. A protein binding to the probe was greatly increased in the anti-IgM treated extracts, and this binding was competed by cold oligonucleotide containing a consensus AP1 motif but not by an oligonucleotide containing the NFAT motif (Fig 4A). These results confirm that BCR activation in BJAB cells results in strongly increased nuclear AP1 activity.

We next determined if the Zp AP1-like motif located between -123 to -129 (TGAGCCA versus the AP1 consensus binding sequence TGAGTCA[41]), can bind AP1 in the presence or absence of the adjacent NFAT motif. A previously published paper did not find AP1 binding to this motif [42]; however, this study did not use probes that also contain the adjacent NFAT motif. As shown in Fig 4B, when nuclear extracts from untreated or anti-IgM treated BJAB cells were incubated with a longer oligonucleotide probe that contains both the NFATc1 and ZIIIA motifs, an additional (larger) protein complex bound to the larger probe, and this complex was found only in the anti-IgM treated cells. Furthermore, this larger complex was competed away with both an unlabeled oligonucleotide containing the consensus AP1 motif, and an oligonucleotide containing the consensus NFAT motif. In addition, the anti-IgM dependent complex was super-shifted by pre-incubation with an anti-cFos (AP1) antibody (Fig 4C) but not an anti-XBP1 antibody. These results confirm that BCR stimulation of BJAB cells allows AP1 to bind to the Zp ZIIIA motif in an NFATc1-dependent manner, and that this only occurs on the Zp-V3 version of the promoter.

### NFATc1 and cFos synergistically activate Zp-V3 but not Zp-P

**Fig 5:**
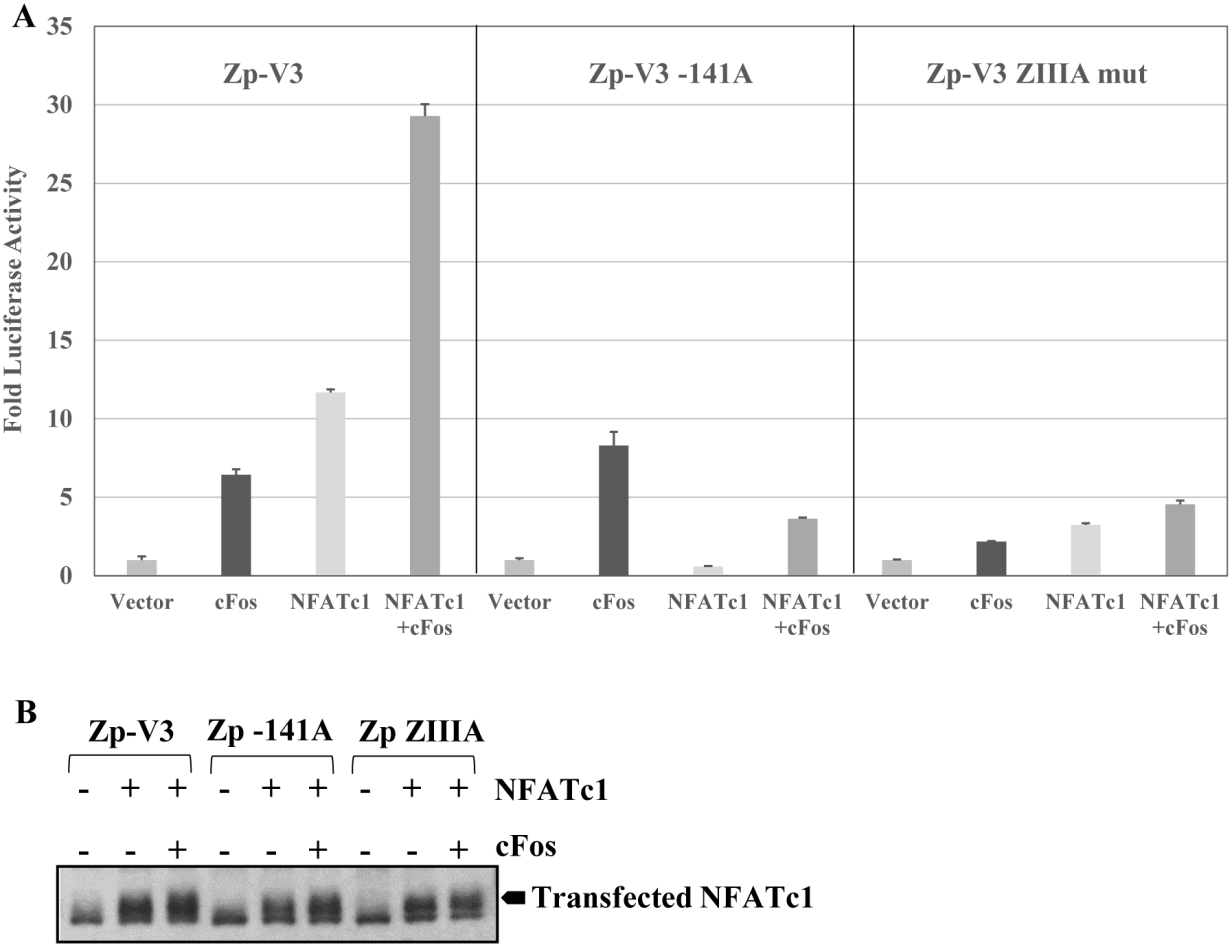
NFATc1 and cFos cooperate to activate the Zp-V3. **(A)** EBV-negative BJAB cells were transfected with the wildtype Zp-V3 luciferase vector, the Zp-V3-141A, or the Zp-V3 ZIIIA mutant promoter luciferase constructs, with or without plasmids expressing cFos and/or NFATc1 as indicated. The fold increase in luciferase activity induced by co-transfection with cFos or NFATc1 vectors for each promoter construct (relative to the vector control, set as 1) is shown. Error bars indicate standard deviation. **(B)** Levels of transfected NFATc1 in each condition were determined by immunoblot.

Given that AP1 is recruited to the Zp-V3 ZIIIA site in an NFATc1-dependent manner, we next asked if the combination of NFATc1 and cFos synergistically activates the Zp-V3 promoter in reporter gene assays. As shown in Fig 5A, the combination of cFos and NFATc1 activated the Zp-V3 luciferase construct much more effectively than either cFos or NFATc1 alone, although the level of transfected NFATc1 was similar with or without co-transfected cFos (Fig 5B). Furthermore, mutation of either the -141 NFAT motif, or the ZIIIA motif, greatly decreased the ability of the NFATc1/cFos combination to activate the promoter. These results confirm that NFATc1 and cFos collaborate to activate the Zp-V3 promoter by binding to the -141 NFAT and ZIIIA sites, respectively.

### The EBV protein LMP2A also activates Zp-V3 through the NFATc1 and ZIIIA sites

**Fig 6:**
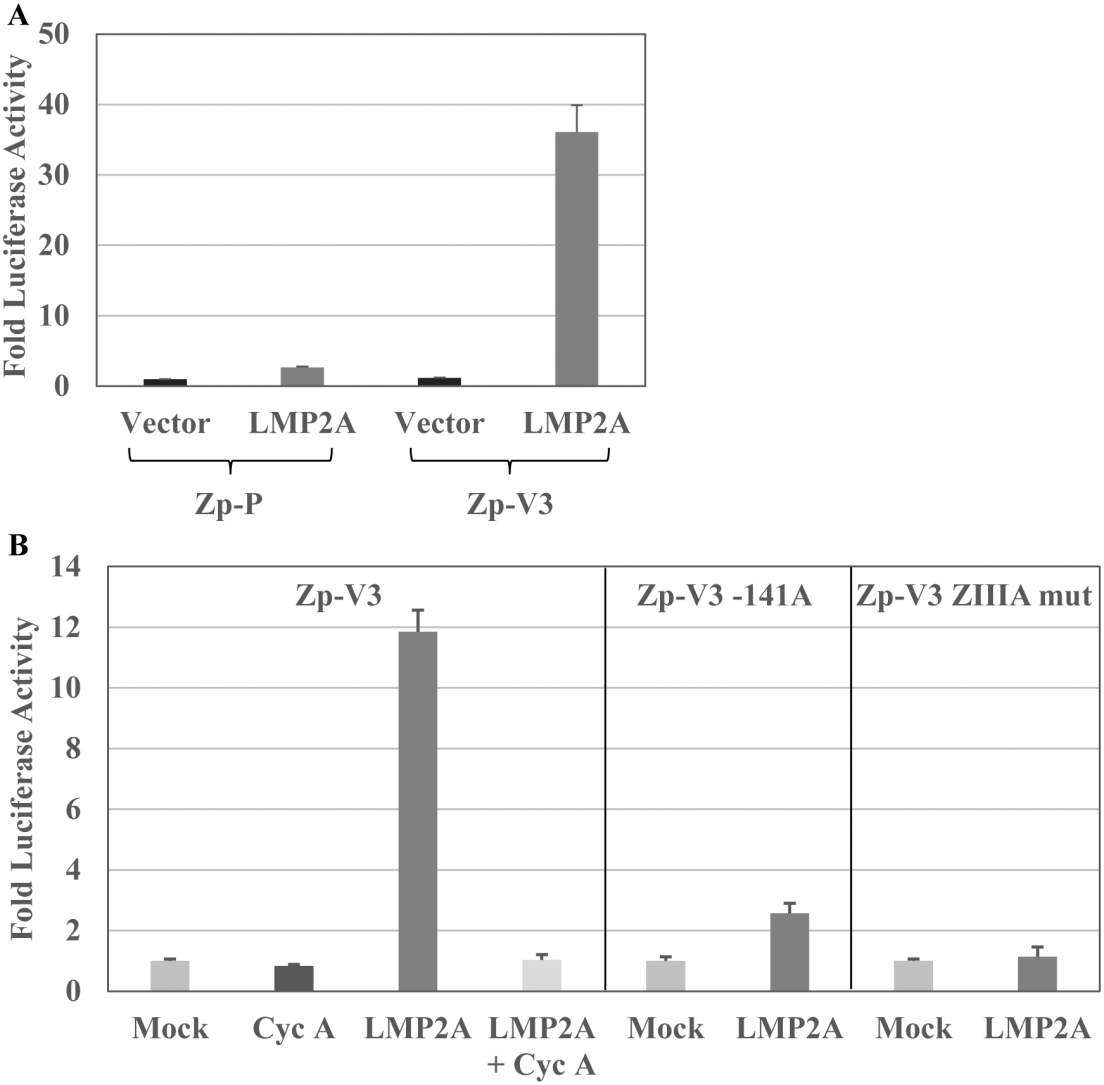
LMP2A, a BCR mimic, activates the Zp-V3 via the NFAT and ZIIIA binding sites. **(A)** EBV-negative BJAB cells were transfected with the Zp-P or Zp-V3 luciferase vector with or without a plasmid expressing LMP2A. The fold increase in luciferase activity induced by co-transfection with the LMP2A vector for each promoter construct (relative to the untreated vector control, set as 1) is shown. Error bars indicate standard deviation. **(B)** BJAB cells were transfected with wildtype Zp-V3 luciferase vector, the Zp-V3-141A, or the Zp-V3 ZIIIA mutant promoter luciferase constructs, with or without a plasmid expressing LMP2A (in the presence or absence of cyclosporine A treatment) as indicated. The fold increase in luciferase activity induced by co-transfection with the LMP2A vector for each promoter construct (relative to the untreated vector control, set as 1) is shown.

The EBV-encoded protein LMP2A mimics constitutively active BCR signaling to enhance B cell survival and proliferation [43–50], and in some, but not all, studies has been reported to activate Zp [51]. To determine if LMP2A can activate the Zp-V3 form of Zp, BJAB cells were transfected with the Zp-V3 or Zp-P reporter gene construct in the presence or absence of a co-transfected LMP2A expression vector. As shown in Fig 6A, LMP2A expression greatly enhanced the activity of the Zp-V3 promoter, but only slightly increased that of the Zp-P promoter. This activation of the Zp-V3 promoter was abolished by mutation of either the NFATc1 or ZIIIA binding motifs (Fig 6B). Furthermore, treatment of cells with the NFAT inhibitor cyclosporine also prevented LMP2A from activating the Zp-V3 promoter. Together these results suggest that, similar to the effect of the authentic BCR, LMP2A signals through NFATc1 to increase Zp-V3 but not Zp-P activity.

### Altering the -141 Zp nucleotide in the context of the intact EBV genome strongly affects lytic protein expression in newly infected B cells

**Fig 7:**
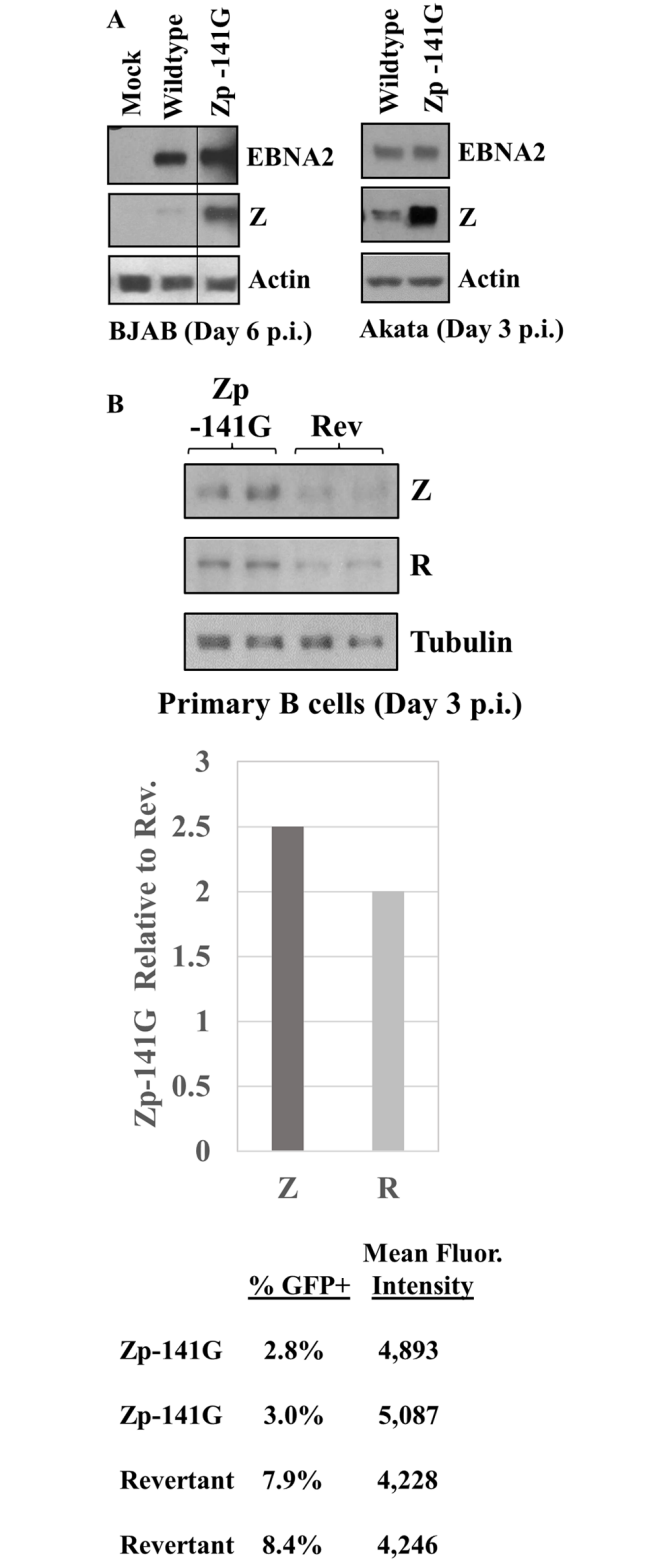
Altering the Zp-P -141 nucleotide to that of the Zp-V3 nucleotide in the context of the intact B95.8 viral genome increases lytic protein expression in newly infected B cells. The -141 residue in the Zp-P of the B95.8 2089 bacmid (encoding GFP) was altered to that of Zp-V3, and infectious viral particles were produced as described in the methods. A revertant construct (Rev), in which the Zp-V3–141 nucleotide was switched back to that of Zp-P, was also constructed. **(A)** Equal titers of wildtype (WT) B95.8, or the Zp -141G mutant, were used to infect EBV-negative BJAB cells (left panel) or EBV-negative Akata cells (right panel). Immunoblots were performed at the time points indicated after infection to detect the EBV protein EBNA2 (as a control for equal amount of EBV infection), Z, and actin (loading control). **(B)** Normal primary peripheral B cells were infected with the Zp mutant or revertant virus as indicated and harvested after three days. In the top panel extracts from unsorted cells were immunoblotted for Z, R (BRLF1), and loading control tubulin. The middle panel quantifies the results of the top panel using Image Studio Lite software to normalize the levels of Z and R expression relative to tubulin expression in each cell type. Results are presented as the ratio of Z and R protein in cells infected with the Zp-141G virus relative to Z and R expression in cells infected with the revertant (wildtype) virus. In the bottom panel, the percent GFP+ cells and mean fluorescence intensity of the cells on day 3 (measured by flow cytometry) is shown.

We next asked if altering a single basepair of the Zp sequence (-141) in the context of the intact approximately 172 Kbp type 1 B95.8 strain EBV genome (which has Zp-P) is sufficient to change the lytic phenotype of the virus. As shown in Fig 7A, B95.8 virus containing the mutated Zp -141 nucleotide (Zp-V3 form) expressed much more Z protein than the WT virus following infection of two different EBV-negative Burkitt lines, BJAB and Akata, although similar levels of the latent EBV protein, EBNA2, were expressed in each cell type.

To determine if the B95.8 Zp-V3 mutant is also more lytic following infection of normal B cells, we infected primary peripheral B cells with the mutant Zp-V3 virus or virus in which the mutation had been reversed back to the WT sequence (revertant virus) using an MOI of 0.1. Since both viruses have the GFP gene inserted into their genomes, we used GFP flow cytometry analysis to examine the number of cells infected with each virus and the level of GFP expression per cell. Although a larger number of B cells (approximately 8% of cells) were infected with the revertant (WT) virus in comparison to the Zp-V3 mutant virus (approximately 3% of cells), the level of Z expression assessed by immunoblot on day 3 after infection was greater in the Zp-V3 mutant infected cells (Fig 7B). Together, these results confirm that a single nucleotide alteration of the BZLF1 promoter is sufficient to confer enhanced lytic gene expression following EBV infection of either primary B cells or Burkitt lymphoma cells.

### The Zp-V3 variant promotes early and late lytic EBV gene expression in stably infected Mutu Burkitt cells treated with the NFAT inducing agent, ionomycin

**Fig 8:**
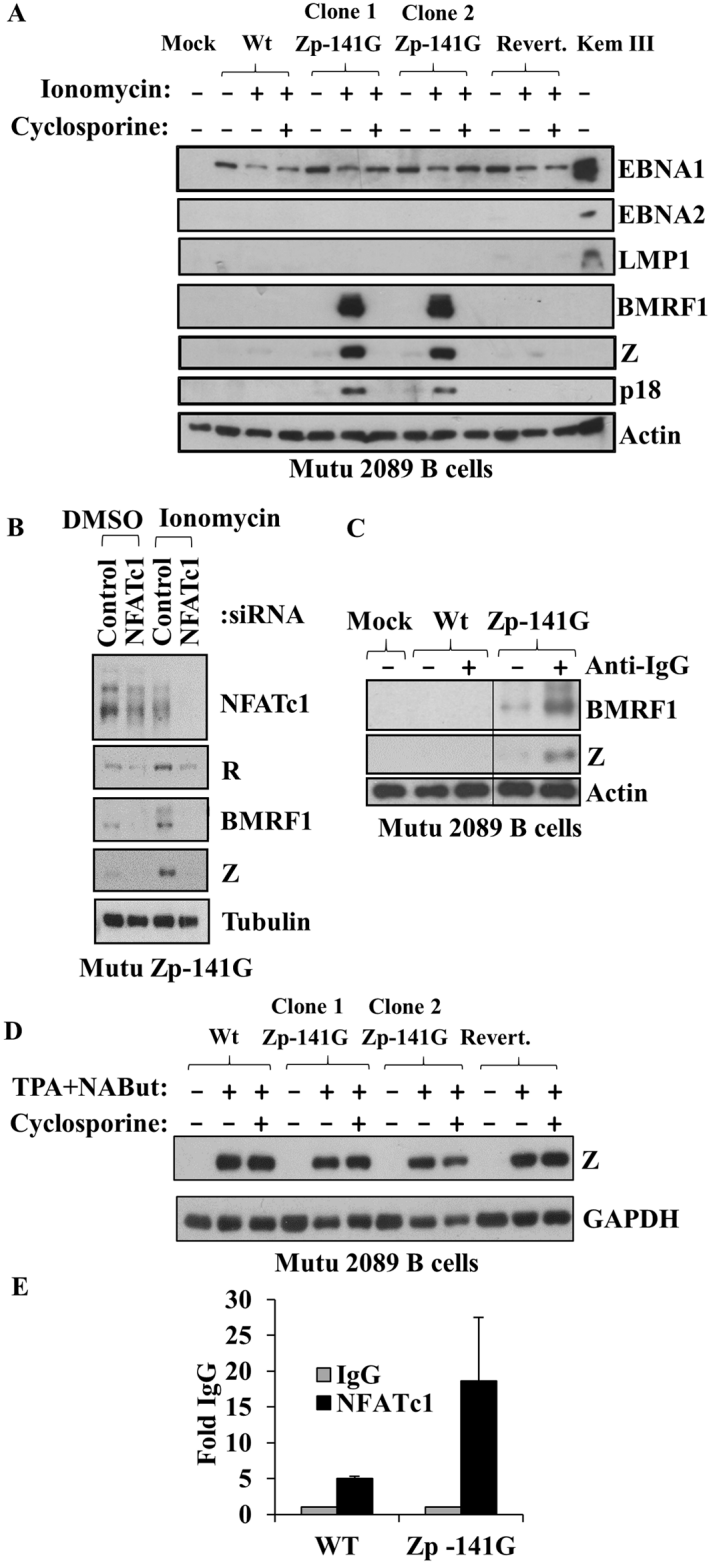
Converting the -141 Zp nucleotide in the intact B95.8 genome to the Zp-V3 nucleotide increases lytic protein expression in stably infected Burkitt cells. **(A)** EBV-negative Mutu B cells were infected with wildtype, Zp mutant, or revertant B95.8 (2089) viruses as indicated, and stably selected with hygromycin B for two months. Two different independently selected lines for each virus were then treated for two days with or without ionomycin (in the presence or absence of cyclosporine), and immunoblots were performed to detect EBNA1, EBNA2, LMP1, Z, BMRF1 (early lytic protein), p18 (late lytic protein), and actin. Kem III cell extract was included as a positive control for EBNA1, EBNA2, and LMP1. **(B)** Mutu cell lines containing Wt or Zp mutant viruses were nucleofected with control siRNA or NFATc1 siRNA. Ionomycin or DMSO control was added after 48 hours, and cells harvested 72 hours post-infection. Immunoblots were performed to detect NFATc1, R, BMRF1, Z, and tubulin (loading control). **(C)** Mutu cell lines containing Wt or Zp mutant viruses (or mock infected cells) were treated with or without anti-IgG for two days and immunoblots performed to detect BMRF1, Z, and actin (loading control). **(D)** Mutu cell lines containing Wt, Zp mutant, or revertant viruses were treated with or without TPA plus sodium butyrate (NaBut) (in the presence or absence of cyclosporine) for two days and immunoblots performed to detect Z expression and GAPDH (loading control). **(E)** ChIP assays were performed using Mutu cell lines containing Wt or Zp mutant viruses treated for three hours with ionomycin. Formaldehyde-fixed cell extracts were immunoprecipitated with control anti-IgG or NFATc1 antibody. qPCR using primers for the EBV Z promoter was performed; results shown are expressed as the amount of Zp complexed to NFATc1 ab relative to the control IgG ab. Data represent three independent experiments.

The expression of early and late lytic EBV genes in B cells requires that the incoming (non-methylated) viral genome becomes highly methylated (since Z preferentially binds to and activates methylated viral promoters) [52–54], and does not occur until at least 2 weeks after EBV infection [53]. To determine if the Zp-V3 sequence enhances the ability of B95.8 virus to lytically reactivate in stably infected cell lines, we infected EBV-negative Mutu cells (derived from a Burkitt lymphoma) with WT, revertant, or Zp-V3 mutant B95.8 viruses, and used hygromycin selection to obtain stably infected cell lines. As shown in Fig 8A, stably infected Mutu cell lines all had type I EBV latency (EBNA1-pos, LMP1-neg, EBNA2-neg), independent of whether cells were infected with the WT, revertant or Zp-V3 mutant viruses. However, when treated with the NFAT-inducing agent, ionomycin, Mutu cells infected with the Zp-V3 mutant had much more Z protein expression, as well as increased expression of an early lytic protein (BMRF1) and a late lytic viral protein (VCA-p18), compared to cells infected with the WT or revertant viruses. Furthermore, the ability of ionomycin treatment to activate lytic EBV protein expression was reversed by cyclosporine treatment (Fig 8A) or NFATc1 siRNA (Fig 8B), confirming that its effect is at least partially mediated through activated NFAT. Likewise, the ability of anti-IgG mediated BCR activation to induce lytic gene expression was increased in Mutu cells infected with the Zp-141G virus (Fig 8C). In contrast, cells infected with the mutant, wildtype, or revertant viruses all induced similar levels of Z expression when treated with the combination of phorbol-12-myristate-13-acetate (TPA) and sodium butyrate (NaBut), and the effect of TPA/sodium butyrate was not reversed by cyclosporine (Fig 8D). Thus, the Zp-V3 variant specifically confers increased Z expression in response to NFAT-inducing agents, and does not globally increase Z expression in response to all lytic inducing agents.

To confirm that NFATc1 binds more efficiently to the Zp-V3 form of the Z promoter in EBV-infected cells *in vivo*, we performed ChIP assays in ionomycin-treated Mutu cells infected with the wildtype or Zp-141G B95.8 viruses (Fig 8E). Consistent with the *in vitro* binding assays, these results showed that endogenous NFATc1 binds more strongly to the Z promoter of the Zp-141G (Zp-V3 type) virus in comparison to the wildtype (Zp-P type) virus.

### The Zp-V3 B95.8 mutant is not defective for transforming primary B cells

**Fig 9:**
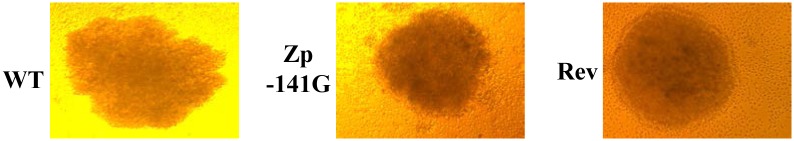
The Zp-V3 B95.8 mutant is not defective for transforming primary B cells. Primary peripheral B cells were infected with wildtype, Zp-141G mutant, and revertant virus (MOI 0.25) and maintained for 21 days. 10/10 wells had colony outgrowth for each virus. Representative bright field images from each strain are shown.

Since excessively lytic EBV infection could be incompatible with the establishment of long-term viral latency and B cell transformation [55], we determined if the Zp-V3 mutant B95.8 virus can transform primary B cells *in vitro*. Purified adult peripheral B cells were infected with the WT or Zp-V3 mutant viruses using 0.25 infectious EBV unit per cell, and the percentage of wells containing lymphoblastoid outgrowths at day 21 post-infection was examined. 10/10 wells infected with either mutant, wildtype or revertant viruses had such outgrowths, suggesting that the Zp-V3 mutant has similar transforming capacity as the WT B95.8 strain virus, at least when using an MOI of 0.25 (Fig 9). Therefore, the enhanced lytic protein expression that occurs following infection of B cells with viruses containing this form of the Z promoter is not sufficient (at least in the context of B95.8 strain EBV) to inhibit viral transformation of primary B cells.

### Zp-V3 is over-represented in type 1 EBV-infected Burkitt lymphomas

The frequency of the Zp-V3 variant in non-malignant tissues is variable depending upon the EBV type (type 1 versus type 2), and geographic region. Interestingly, type 2 EBV is most common in areas of the world where malaria and Burkitt lymphoma are endemic, and the Zp-V3 form of the BZLF1 promoter is present in all type 2 EBV genomes sequenced to date [56,57]. In contrast, Zp-V3 is relatively rare in type 1 EBV, except for type 1 EBV genomes isolated from Asian EBV strains [56]. Although the Zp-V3 variant has been shown to be over-represented in type 1 EBV genomes isolated from NPCs in China, and in AIDs-related lymphomas in Italy (relative to its frequency in non-malignant samples obtained from humans living in the same geographic regions [22,23]), whether the Zp-V3 variant is over-represented in type 1 EBV genomes in Burkitt lymphomas (BLs) is not yet known.

To investigate this, we examined the Zp status of EBV-infected BLs obtained from Africa or South America [56,58–60] (detailed in S1 Table) versus EBV genomes derived from non-malignant samples from the same geographic regions (spontaneous LCL samples and infectious mononucleosis samples [56,60]; detailed in S2 Table). Zp sequence alignments from malignant and normal tissues for previously unanalyzed promoter variant sequences are shown in S6 Table.

Type 2 EBV genomes were similarly represented in the Burkitt lymphoma and non-malignant samples (Table 1), and as expected all type 2 EBV genomes had the Zp-V3 form of Zp (Table 2). Importantly, we found that type 1 EBV genomes in BLs are much more likely to contain the Zp-V3 variant of Zp (37%) versus type 1 EBV genomes obtained from non-malignant samples (4%); p <0.003 by Fisher’s exact test (Table 2). This result reveals that Zp-V3 containing T1 EBV is over-represented (and likely selected for) in EBV-infected Burkitt lymphomas.

**Table 1:** Type 1 and Type 2 EBV frequency in EBV-positive Burkitt lymphomas in Africa and South America.

Sample Type	Type 1 EBV	Type 2 EBV
Burkitt lymphomas	84% (38/45)	16% (7/45)
Non-malignant	81% (25/31)	19% (6/31)

The frequency of type 1 versus type 2 EBV in EBV-positive Burkitt lymphomas was compared to the frequency in non-malignant tissues (spontaneous LCLs (sLCLs) or PBMCs from infectious mononucleosis (IM) patients) obtained from Africa and South America as detailed in S1 and S2 Tables.

**Table 2:** Zp-V3 is over-represented in type 1 EBV-positive Burkitt lymphomas in Africa and South America.

Sample Type	Zp-V3 in type 1 EBV	Zp-V3 in type 2 EBV
Burkitt lymphomas	37% (14/38)	100% (7/7)
Non-malignant	4% (1/25)	100% (6/6)
p-value (BL vs non-malignant)	<0.003	NS

The frequency of Zp-V3 in type 1 and type 2 EBV-positive Burkitt lymphomas from Africa and South America was compared to the frequency of Zp-V3 in non-malignant tissues (spontaneous LCLs (sLCLs) or PBMCs from infectious mononucleosis (IM) patients) obtained from Africa and South America as detailed in S1 and S2 Tables. Fisher’s exact (2-sided) test was performed.

### Zp-V3 is over-represented in EBV-infected gastric cancers

Up to 10% of gastric cancers worldwide are EBV-infected [61–64] and the genomes of a number of EBV-infected gastric cancers are now available in Genbank and the TCGA database. To determine whether the Zp-V3 variant is also over-represented in gastric carcinomas, we examined the Zp status of 41 type 1 EBV-infected gastric carcinomas obtained worldwide [65–68], versus 113 type 1 EBV genomes derived from non-malignant samples [22,56,69–71] (including spontaneous LCL samples, infectious mononucleosis samples, saliva from healthy individuals, and EBV genome sequences detected in non-tumor control tissues (of any kind) in the whole exome sequence (WXS) TCGA database) as detailed in S3–S5 Tables. Zp sequence alignments from malignant and normal tissues for previously unanalyzed promoter variant sequences are shown in S6 Table. Since the Zp-V3 variant is much more common in type 1 EBV isolates from Asia, and gastric carcinoma is also more common in Asia, we also compared the frequency of Zp-V3 in gastric carcinomas versus non-malignant tissues isolated from samples obtained from either Asian (known Asian individuals or samples obtained from Asian countries) or presumed non-Asian individuals (known Caucasian individuals, or samples obtained from non-Asian countries).

As shown in Table 3, the Zp-V3 variant is significantly over-represented in type 1 EBV-positive gastric cancers worldwide (44% of tumors) relative to its frequency in non-malignant tissues (19%) (p < 0.003 using a Fisher’s exact test). When only samples from Asian (or presumed Asian) patients are examined, Zp-V3 type 1 EBV is still over-represented in gastric tumors (68%) relative to the non-malignant samples (27%) (p < 0.001 using a Fisher’s exact test) (Table 4). Likewise, when only samples from presumed non-Asian patients are examined, Zp-V3 type 1 EBV is still over-represented in gastric tumors (17%) relative to the non-malignant samples (0%) (p < 0.03 using a Fisher’s exact test) (Table 5). Thus, the Zp-V3 variant may also increase the risk of developing EBV-positive gastric cancers.

**Table 3:** Frequency of Zp-V3 in type 1 EBV positive gastric carcinomas. Total gastric carcinomas.

Sample Type	Frequency of Zp-V3
**Gastric Carcinomas**	44% (18/41)
**non-malignant**	19% (21/113)
**p-value (GC vs non-malignant)**	<0.003

The frequency of Zp-V3 in type 1 EBV-positive gastric carcinomas was compared to the frequency of Zp-V3 in type 1 non-malignant tissues (including spontaneous LCLs (sLCLs), PBMCs from healthy or infectious mononucleosis (IM) patients, saliva from healthy individuals, and contaminating EBV genomes from the TCGA database, as detailed in S3–S5 Tables) using results from all available sequences. Fisher’s exact (2-sided) test was performed.

**Table 4:** Frequency of Zp-V3 in Asian type 1 EBV positive gastric carcinomas.

Sample Type	Frequency of Zp-V3
**Gastric carcinomas**	68% (15/22)
**non-malignant**	27% (21/77)
**p-value (GC vs non-malignant)**	<0.001

The frequency of Zp-V3 in type 1 EBV-positive gastric carcinomas was compared to the frequency of Zp-V3 in type 1 non-malignant tissues (including spontaneous LCLs (sLCLs), PBMCs from healthy or infectious mononucleosis (IM) patients, saliva from healthy individuals, and contaminating EBV genomes from the TCGA database, as detailed in S3–S5 Tables), using results from Asian or presumed Asian individuals (i.e., samples obtained in Asian countries). A Fisher’s exact (2-sided) test was performed.

**Table 5:** Frequency of Zp-V3 in non-Asian type 1 EBV positive gastric carcinomas.

Sample Type	Frequency of Zp-V3
**Gastric Carcinomas**	17% (3/17)
**non-malignant**	0% (0/36)
**p-value (GC vs non-malignant)**	< 0.03

The frequency of Zp-V3 in type 1 EBV-positive gastric carcinomas was compared to the frequency of Zp-V3 in type 1 non-malignant tissues (including spontaneous LCLs (sLCLs), PBMCs from healthy or infectious mononucleosis (IM) patients, saliva from healthy individuals, and contaminating EBV genomes from the TCGA database, as detailed in S3–S5 Tables), using results from known Caucasian, or presumed non-Asian individuals (i.e., results obtained from the USA, Italy and Australia). A Fisher’s exact (2-sided) test was performed.

### Zp-V3 is over-represented in LCLs derived from EBV-infected breast milk of Kenyan women that had malaria during pregnancy

Finally, given the recent finding that infectious EBV in the breast milk of mothers that had malaria during pregnancy may serve as a source of EBV transmission to neonates [17], we examined whether LCL lines derived from the breast milk of these mothers have a high prevalence of the Zp-V3 variant. For this analysis, we sequenced the Z promoter in the EBV genomes of LCLs transformed by breast-milk-derived EBV from 10 different Kenyan mothers that had malaria during pregnancy; we then compared the frequency of this variant in the breast-milk derived LCLs to that observed in 13 different spontaneous EBV-infected LCLs derived from the blood of healthy Kenyan individuals residing in areas with high frequency malaria transmission (Table 6). We excluded LCLs infected with type 2 EBV (6/19 of the spontaneous LCLs in healthy Kenyan donors, versus 0/10 LCLs derived from breast milk) from this analysis since all type 2 isolates are known to contain the Zp-V3 variant.

**Table 6:** Zp-V3 is over-represented in breast-milk derived LCLs from Kenyan mothers that had malaria during pregnancy relative to LCLs derived from the blood of healthy Kenyans.

Type 1 LCLs	Zp-V3	Zp-P
Peripheral blood from healthy adults in Kenya	0	13
Breast milk from malaria patients in Kenya	10	0

Surprisingly, all 10 of the breast milk-derived LCLs contained the Zp-V3 variant, and all also had type 1 EBV. In comparison, 0/13 of type 1 EBV-infected LCLs derived from the blood of healthy Kenya donors contained the Zp-V3 variant (p < 0.001). These results suggest that Zp-V3 containing EBV strains may be more prone to lytic reactivation in breast milk than Zp-P containing strains.

## Discussion

Latent EBV infection transforms primary B cells *in vitro* and clearly contributes to the development of a number of EBV-associated human malignancies. However, since the most transforming form of EBV latency (type III) is sufficient to induce proliferation and survival of B cells, whether lytic EBV infection also contributes to EBV-associated malignancies is less clear. Here we have examined whether a cancer-associated polymorphism of the viral BZLF1 immediate-early promoter affects lytic EBV gene expression. We demonstrate that this cancer-associated promoter variant contains an NFATc1 binding motif not present in the prototype promoter, and has enhanced lytic gene activation in response to BCR stimulation. Indeed, we find that altering only a single basepair of the BZLF1 promoter (to confer NFATc1 binding) in the context of the intact type 1 EBV genome is sufficient to increase the amount of lytic EBV protein expression in B cells. In addition, we show that the presence of high EBV titers in the breast milk of malaria-infected Kenyan women (measured by the ability of the milk to transform B cells into LCLs *in vitro*) is highly associated with the presence of the Zp-V3 variant in type 1 EBV strains. Importantly, we also show for the first time that Zp-V3 containing type 1 EBV is likewise over-represented in both Burkitt lymphomas, and gastric carcinomas, relative to non-malignant control samples. Together, the studies presented here suggest that enhanced lytic EBV gene expression increases the likelihood of at least some types of EBV-associated malignancies in humans.

BCR stimulation has been known for some time to induce lytic EBV reactivation in many EBV-infected Burkitt cell lines, but the major BCR effect was previously reported to be mediated through post-translational modification of MEF2 family members that can also bind to the BZLF1 promoter [25,72]. However, many of these previous studies used the Zp-P form of the promoter, which is found in the prototype laboratory EBV strain (B95.8). While a previous paper reported that BCR-mediated stimulation of the Zp-P promoter is largely dependent on a feed-forward loop in which the BZLF1 protein binds to and activates its own promoter (once a small amount of Z gene expression has been stimulated by cellular transcription factors such as MEF2) [39], we show that BCR stimulation directly and strongly activates the Zp-V3 version of the promoter without the requirement for concomitant Z protein expression. We also found that the AP1-like “ZIIIA” motif is required for BCR-mediated activation of Zp-V3, and binds AP1 in an NFAT-dependent manner. In contrast, previous studies using the Zp-P promoter variant found that the ZIIIA motif primarily serves as a binding site for the Z protein itself (rather than AP1 binding) [39,42,73]. Since the NFATc1 binding site in the Zp-V3 variant overlaps the “ZIC” motif previously reported to be important for TPA-induced activation of the Zp-P variant, we cannot exclude the possibility that additional transcription factors can also regulate Z transcription through this motif in one or both promoter variants.

Given the long-appreciated epidemiologic association of malaria infection with EBV-induced Burkitt lymphomas in Africa, and the growing evidence that malaria increases the amount of lytic EBV in co-infected patients, we asked if the presence of infectious EBV in the breast milk of Kenyan mothers that had malaria during pregnancy correlates with the presence of the Zp-V3 form of the EBV promoter. This seems to be the case, since 10/10 of the type 1 EBV LCL lines derived from infectious EBV in the breast milk of these women had this form of the EBV promoter, versus only 0/13 type 1 LCL lines derived from the blood of healthy Kenyan patients in high malaria regions.

B cells in breast milk are more highly activated, and much more likely to differentiate into plasmablasts or plasma cells, than the B cells in peripheral blood [74]. The known propensity of EBV to lytically reactivate in antigen-stimulated plasma cells, and our finding that the Zp-V3 variant is particularly responsive to BCR stimulation, may explain why the Zp-V3 variant is highly over-represented in LCLs derived from infectious EBV in the breast milk of Kenyan mothers that had malaria during pregnancy. Our results suggest that Zp-V3-containing EBV strains may be more easily transferred to nursing neonates than EBV strains containing the Zp-P variant, particularly if the breast milk does not contain enough maternal EBV-neutralizing antibodies to block infection of the neonate (as may occur in malaria). If so, very early EBV infection in the context of an immature immune system may predispose infants to developing Burkitt lymphoma. Clearly, however, larger prospective studies are needed to confirm that Zp-V3 containing EBV strains are more likely than Zp-P containing strains to produce infectious viral particles in breast milk, and/or to be transmitted from breast milk to nursing infants.

Whether the Zp-V3 variant also confers greater Z expression in epithelial cells is not yet clear. However, since we previously showed that B95.8 EBV-infected gastric AGS cells (one of the few epithelial cell lines easily infected with this strain) are remarkably lytic [75], the Zp-P promoter variant is clearly quite active in this cell type. In addition, our finding here that KLF4, a cellular transcription factor required for lytic EBV reactivation during epithelial cell differentiation [24], activates the Zp-P and Zp-V3 variants with similar efficiency suggests that the Zp-P and Zp-V3 versions of the promoter may have similar activity in infected epithelial cells. Furthermore, a recent study showed that similar levels of EBV are contained in the saliva of United Kingdom college students infected with Zp-V3 containing EBV strains versus Zp-P containing strains [56]. Thus, over-representation of the Zp-V3 variant in nasopharyngeal carcinomas [22,23] and gastric carcinomas (shown here) may reflect increased hematogenous delivery of B-cell derived infectious EBV particles to nasopharyngeal and gastric epithelial cells.

There are two major strains of EBV (referred to as type 1 and type 2), and essentially all type 2 EBV strains carry the Zp-V3 version of the BZLF1 promoter, whereas only a minority of the type 1 strains carry this version [56,57]. The major phenotypic differences between the type 1 and type 2 strains of EBV are thought to reflect differences in the sequences of the essential latent transforming genes, EBNA2 and EBNA3A/3C [76–80]. Although type 2 EBV transforms B cells *in vitro* less efficiently than type 1 EBV [81], there is no evidence that type 2 EBV is less competent for causing EBV-associated Burkitt lymphomas in humans [82]. Indeed, type 2 strain EBV infection is particularly common in regions of Africa that have high rates of Burkitt lymphoma, although even in these regions type 1 strain is still more common [82]. Given the many differences between type 1 and type 2 EBV, it is important to stress that our studies here demonstrate that the Zp-V3 version of the Z promoter is more lytic than the Zp-P version in the context of a type 1 EBV genome (B95.8 strain). Thus our results are not confounded by other potential important differences between the two strains due to alterations in the EBV latency proteins. Nevertheless, since we show here that the Zp-V3 promoter variant produces more lytic EBV reactivation than the Zp-P variant in response to BCR activation, and all type 2 EBV strains sequenced to date contain the Zp-V3 version of the promoter, it is interesting to speculate that increased lytic reactivation by type 2 EBV strains may partially compensate for the less transforming phenotype (at least *in vitro*) in terms of the ability of type 2 strains to promote Burkitt lymphomas.

An interesting unanswered question is why EBV has evolved to have at least two different BZLF1 promoter variants. It is possible that the Zp-P variant common in the type 1 strain ensures that the virus can establish long-term latency in B cells, whereas the Zp-V3 variant that is universally present in type 2 strains (as well as some type 1 strains) increases the efficiency of lytic virus reactivation and helps ensure efficient horizontal transmission. Many individuals (particularly when immunosuppressed) are co-infected with type 1 and type 2 EBV strains [83], and the breast milk of Kenyan women that had malaria during pregnancy was found to commonly contain both types of EBV [17]. Furthermore, a number of recombinant EBV genomes containing portions of both type 1 and type 2 EBV have now been found in tumors and LCLs [57], suggesting that individual B cells may be simultaneously infected with both types of EBV. In B cells infected with more than one virus type, Z protein transcribed from the Zp-V3 promoter could simultaneously induce reactivation of the Zp-P carrying strain, since once made, Z can auto-activate either form of the Z promoter.

However, an important finding in our studies was that type 1 EBV strains containing the Zp-V3 BZLF1 promoter variant are particularly over-represented in Burkitt lymphomas relative to either Zp-P containing type 1 EBV or Zp-V3 containing T2 EBV strains. Therefore, we speculate that Zp-V3 containing type 1 strains may be especially transforming because they incorporate both the increased lytic activity of Zp-V3 (usually type 2)-containing EBV strains, and the enhanced transforming functions of type 1 EBV strains. Of note, although Zp-V3 incorporation into otherwise type 1 EBV genomes may result from recombination between type 1 and type 2 EBV strains, we identified some Burkitt lymphomas and gastric carcinomas in which only the Zp -141 nucleotide was switched to the Zp-V3 form of the promoter, and the other two variant nucleotides were the same as the Zp-P variant. In contrast, we found no tumors in which one of the other Zp-P specific nucleotides was mutated to the Zp-V3 variant without mutation of the Zp -141 nucleotide. This result suggests that in some instances, mutations which specifically convert the Zp-P -141 sequence to the Zp-V3 sequence may be selected for in EBV-infected tumors in the absence of recombination. Our results also raise the interesting possibility that certain EBV-associated cancers are particularly common in Asia due to the high frequency of Zp-V3 containing T1 EBV strains in this part of the world.

Finally, our findings suggest that additional studies to examine whether the Zp-V3 version of the BZLF1 promoter is associated with increased lytic EBV infection in humans are warranted. If this proves to be the case, it would have important clinical implications, and would buttress the argument that anti-EBV vaccines that inhibit lytic infection without preventing the establishment of viral latency might be useful for preventing EBV-associated malignancies.

## Materials and methods

### Cell lines and culture

The EBV-negative B cell lines Mutu (a gift from Jeff Sample), BJAB (purchased from ATCC), and Akata (a gift from Kenzo Takada), and EBV-positive Raji (ATCC) and Kem III (a gift from Alan Rickinson and Jeff Sample) were grown in RPMI-1640 media (Gibco). All media was supplemented with 10–15% FBS and 1% penicillin-streptomycin (pen-strep). The epithelial line HEK 293 (from ATCC) was maintained in DMEM (Gibco). Mutu cells infected with the EBV p2089 Bacmid (B95.8) were maintained under selection of 300ug/mL Hygromycin B. Primary human peripheral CD19+ B cells from healthy donors (obtained from Stem Cell Technologies (#70033), who used Institutional Review Board (IRB)-approved consent forms and protocols) were EBV-transformed and grown in RPMI. The NOKs cell line (a gift from Karl Munger) is a telomerase-immortalized normal oral keratinocyte cell line that was established and maintained as previously described [84].

### Drug treatments

Cells were treated with the following drugs for experiments: ionomycin (Calbiochem) at 2.5μg/mL, TPA (Sigma) at 20ng/mL, cyclosporine A (Cell Signaling) at 1μM, sodium butyrate (Sigma) at 3mM, anti-IgM (Southern Biotech) at 10μg/mL, anti-IgG (Sigma I5260) at 10μg/mL. When cyclosporine was added to cells, it was done one hour prior to addition of any other drugs.

### Plasmids and cloning

Plasmid DNA was prepared using the Qiagen Maxiprep kit according to the manufacturer’s instructions. Plasmid pSG5 was obtained from Stratagene. SG5-LMP2A was a gift from Nancy Raab-Traub. pREP-NFAT2 (NFATc1) was a gift from Anjana Rao (Addgene plasmid # 11788). pLX304-FOS-V5 was a gift from William Hahn (Addgene plasmid # 59140) [85]. The promoterless luciferase reporter gene construct pCpGL-basic (a gift from Michael Rehli) was constructed as previously described [86] and contains no CpG motifs in the entire vector. The BZLF1 promoter (-668 to +15, relative to transcription start site) was PCR amplified from the EBV B95.8 (Zp-P) and M81 (Zp-V3) genomes and cloned upstream of the luciferase gene in pCpGL-basic using the SpeI and BglII restriction sites. The primer sequences are as follows: BZLF1 F SpeI 5’-GCGACTAGTAGGTGTGTCAGCCAAAG and BZLF1 R BglII 5’- GCGAGATCTCCGGCAAGGTGCAATG. Zp mutants in the pCpGL luciferase vector were constructed using the Stratagene QuikChange II XL site-directed mutagenesis kit. Primers are as follows: Zp-V3–100 5’- CTAATGTGCCTCATAGACACACCTAAATTTAGCACGTCC and 5’- GGACGTGCTAAATTTAGGTGTGTCTATGAGGCACATTAG; Zp-V3–106 5’- ACAGGCATTGCTAATGTACCTCAGAGACACACCTA and 5’- TAGGTGTGTCTCTGAGGTACATTAGCAATGCCTGT; Zp-V3–141 5’- CTGCCTCCTCCTCTTTTAGAAACTATGCATGAGCC and 5’- GGCTCATGCATAGTTTCTAAAAGAGGAGGAGGCAG; Zp-V3–274 5’- CTCCCCCCTGACCCCCGAACTTAATGAAATCTTGGA and 5’- TCCAAGATTTCATTAAGTTCGGGGGTCAGGGGGGAG; Zp-V3–365 5’- AGATGGACCTGAGCCACCCGCCCCC and 5’- GGGGGCGGGTGGCTCAGGTCCATCT; Zp-V3–460 5’- GGAGGACCCTGATGAAGAAACCAGTCAGGCC and 5’- GGCCTGACTGGTTTCTTCATCAGGGTCCTCC; Zp-V3–525 5’- CGGTGCCCCAGCCACTTGACCCGG and 5’- CCGGGTCAAGTGGCTGGGGCACCG; Zp-P -141 5’- CTGCCTCCTCCTCTTTTGGAAACTATGCATGAGCC and 5’- GGCTCATGCATAGTTTCCAAAAGAGGAGGAGGCAG. The ZIIIA/AP1 Zp-V3 mutant was mutated sequentially, first with Zp-V3 Ap1 mut 1 5’-TTGGAAACTATGCAGAAGCCACAGGCATTGCTAATGTGCCT and 5’-AGGCACATTAGCAATGCCTGTGGCTTCTGCATAGTTTCCAA, then with Zp-V3 AP1 mut 2 5’-TTGGAAACTATGCAGAATTCACAGGCATTGCTAATGTGCCT and 5’-AGGCACATTAGCAATGCCTGTGAATTCTGCATAGTTTCCAA. All constructs were verified by sequencing.

### Construction of EBV mutant genomes

The EBV p2089 Bacmid was a gift from Henri-Jacques Delecluse and contains the complete genome of the B95.8 strain of EBV in addition to a cassette containing the prokaryotic F-factor as well as the green fluorescent protein (GFP) and Hygromycin B resistance genes in the B95.8 deletion as previously described [87]. p2089 is the parental WT Bacmid to all mutants in this study. EBV Zp-P-141G Bacmid was constructed using the GS1783 E. coli–based En Passant method previously described [88] to change the -141 nucleotide in the B95.8 Zp to Variant 3 Zp sequence. Subsequently a revertant Bacmid, designated EBV Zp-P-141G.REV was constructed, reverting the altered nucleotide to wildtype B95.8 sequence. Finally, the Chloramphenicol cassette in the F-factor of each WT, Zp-P-141G, and Zp-P-141G.REV Bacmids was replaced with Kanamycin. Kanamycin resistance facilitated the transfer of all Bacmids to the Chloramphenicol-resistant BM2710 E. coli [89] used for infection of 293 cells. The integrity of each Bacmid was confirmed by analyzing the restriction digestion patterns with multiple enzymes. Furthermore, all mutations were confirmed by high fidelity PCR amplification and sequencing of the mutated junctions. The list of primers used for generation and confirmation of all mutants is as follows. Zp.T-141C Primer 1 5’- TGAGGTACATTAGCAATGCCTGTGGCTCATGCATAGTTTCCAAAAGAGGAGGAGGCAGTTTTAGGGATAACAGGGTAATCGATTT, Zp.T-141C Primer 2 5’-CTTATTTTAGACACTTCTGAAAACTGCCTCCTCCTCTTTTGGAAACTATGCATGAGCCACAGCCAGTGTTACAACCAATTAACC, Zp.T-141C.REV Primer 1 5’-TGAGGTACATTAGCAATGCCTGTGGCTCATGCATAGTTTCTAAAAGAGGAGGAGGCAGTTTTAGGGATAACAGGGTAATCGATTT, Zp.T-141C.REV Primer 2 5’-CTTATTTTAGACACTTCTGAAAACTGCCTCCTCCTCTTTTAGAAACTATGCATGAGCCACAGCCAGTGTTACAACCAATTAACC, Cam-Ff-Kan Primer 1 5’-CGGGCGTATTTTTTGAGTTATCGAGATTTTCAGGAGCTAAGGAAGCTAAAATGAGCCATATTCAACGGGAAAC, Cam-Ff-Kan Primer 2 5’-CAGGCGTAGCAACCAGGCGTTTAAGGGCACCAATAACTGCCTTAAAAAAATTAGAAAAACTCATCGAGCATC, Zp.-141-Confirm Primer 1 5’-CGGCAAGGTGCAATGTTTAG, and Zp.-141-Confirm Primer 2 5’-GTGTCAGCCAAAGAGGATCA.

### Luciferase assays

BJAB cells were nucleofected using the Amaxa Nucleofector 2b device (Lonza) and program M-013 (with Buffer V) in 12-well dishes with 500 ng of pCpGL-basic promoter construct and 500 ng of vector control, LMP2A, cFos, or NFATc1 plasmid. NOKs were transfected with Lipofectamine 2000 (Thermo-Fisher Scientific). The cells were washed with PBS and harvested in 1× Reporter Lysis Buffer (Promega) at 24–48 h post-nucleofection or transfection. Lysates were subjected to three freeze-thaw cycles, and relative luciferase units were quantified with a BD Monolight 3010 luminometer (BD Biosciences) using Promega luciferase assay reagent. All luciferase assay figures represent two independent experiments, each performed in duplicate.

### Preparation of nuclear extracts

BJAB cells were treated or mock-treated with anti-IgM for 30 minutes or 6 hours and then harvested. The cell pellet was resuspended in 100uL hypotonic buffer A (10mM HEPES-K+ pH7.9, 10mM KCl, 1.5mM MgCl_2_, 0.5mM DTT) in the presence of protease inhibitor cocktail (PIC, Roche) and phosphatase inhibitor cocktail II (Calbiochem), then incubated on ice for 10 min with vortexing. 1uL of 10% NP-40 was added and samples were vortexed to assist in the lysis for up to 1 min. The nuclei were centrifuged at 14,000 RPM for 5 min at 4°C. The nuclear pellets were resuspended in 50uL buffer C (20mM HEPES-K+ pH7.9, 420mM NaCl, 0.2mM EDTA, 1.5mM MgCl_2_, 0.5mM DTT, 25% Glycerol) with PIC. Nuclei were incubated on ice for 40 min, and vortexed periodically. Supernatant containing nuclear protein was collected by centrifuging at 14,000 RPM for 10 min at 4°C and then aliquoted and snap frozen for use in EMSAs.

### EMSA

EMSAs were performed as previously described [90,91]. Consensus binding probes (oligonucleotides) for AP1 (#sc-2501), Ets (#sc-2549), and NFAT (2) (#sc-2577) were obtained from Santa Cruz. All other probes were custom designed and ordered from IDT. Their sequences are as follows: NFAT (1) consensus EMSA 5’- AGAAAGGAGGAAAAACTGTTTCATACAGAAGGCGTT and 5’- AACGCCTTCTGTATGAAACAGTTTTTCCTCCTTTCT; ELK1 consensus EMSA 5’- GGGGTCCTTGAGGAAGTATAAGAAGAAT and 5’- ATTCTTCTTATACTTCCTCAAGGACCCC; Zp-P -155 to -127 EMSA 5’-CCTCCTCCTCTTTTAGAAACTATGCATGA and 5’- TCATGCATAGTTTCTAAAAGAGGAGGAGG; Zp-V3–155 to -127 EMSA 5’-CCTCCTCCTCTTTTGGAAACTATGCATGA and 5’- TCATGCATAGTTTCCAAAAGAGGAGGAGG; Zp-P -155 to -120 EMSA 5’-CCTCCTCCTCTTTTAGAAACTATGCATGAGCCACAG and 5’- CTGTGGCTCATGCATAGTTTCTAAAAGAGGAGGAGG; Zp-V3–155 to -120 EMSA 5’-CCTCCTCCTCTTTTGGAAACTATGCATGAGCCACAG and 5’- CTGTGGCTCATGCATAGTTTCCAAAAGAGGAGGAGG. EMSAs were performed with binding buffer (50 mM KCl, 25 mM Hepes (pH 7.6), 10% glycerol, 1 mM EDTA, 0.5 mM spermidine, 0.5 mM PMSF, and 1 mM DTT) with 2 μg of poly(dI/dC):poly(dI/dC) (Pharmacia) and 2ug BJAB nuclear extract. The protein and binding buffer mixture was allowed to incubate for 5 min at room temperature, and then 20,000 cpm of γ-32P ATP labeled probe were added. The mixture containing labeled probes was allowed to incubate for an additional 20 min. For supershift conditions 1-2ug anti-NFATc1 (Santa Cruz #sc-13033x), anti-XBP1 (Santa Cruz #sc-7160x), anti-cFos (Santa Cruz #sc-52x), or anti-C/EBPα (Santa Cruz #sc-61x) antibodies were added to the protein before addition of radiolabeled probe and allowed to incubate for 20 minutes. For cold competitor conditions 10X excess unlabeled probe was added to the protein before addition of radiolabeled probe and allowed to incubate for 20 minutes.

### Immunoblotting

Immunoblotting was performed as previously described [52]. The following primary antibodies were used: anti-EBNA1 (Santa Cruz #sc-81581), anti-EBNA2 (Abcam #ab90543), anti-LMP1 (Abcam #ab78113), anti-β-actin (Sigma #A5441), anti-GAPDH (Cell Signaling Technology #D16H11), anti-BMRF1 (Millipore #MAB8186), anti-p18 (Thermo Scientific #PA1-73003), anti-BZLF1 (Santa Cruz #sc-53904), anti-R rabbit polyclonal antibody directed against the R peptide (peptide sequence EDPDEETSQAVKALREMAD), anti-NFATc1 (Santa Cruz sc-17834) and anti-tubulin (Sigma T5168). The secondary antibodies used were horseradish peroxidase (HRP)–goat anti-mouse (Thermo Scientific #31430) and donkey anti-goat (Santa Cruz #sc-2056). Image Studio Lite software was used to quantify levels of Z and R relative to loading control tubulin in Fig 7B.

### Derivation of wild-type and mutant EBV-positive 293 cell lines

EBV-positive 293 WT, Zp-P-141G and Zp-P-141G.REV were derived using the BM2710 *E*. *coli*, which can mediate the transfer of intact recombinant DNA into mammalian cells due to expression of the *invasin* gene from *Yersinia pseudotuberculosis* and the *listeriolysin O* gene from *Listeria monocytogenes* [89]. Briefly, Bacmids were electroporated using a 0.1 cm gap cuvette (1.5 kV, 200 Ohms, 25 μF) into BM2710 *E*. *coli* and selected with Kanamycin and Spectinomycin. BM2710 *E*. *coli* containing the respective Bacmid were used to infect EBV-negative 293 cells by co-incubation for 2 hours (approximately 25 bacteria per cell). Cell lines were derived by single-cell cloning and screened for ability to complete the lytic cascade by immunoblotting for viral late protein VCAp18 (product of EBV *BFRF3)* and titering cell-free virus on Raji cells. Cells were selected and maintained with 100–200μg/ml of Hygromycin B.

### Production of infectious virus

Infectious viral particles were produced from 293 cell lines stably infected with the wildtype or mutant B95.8 viruses as previously described [92]. To determine the titer of the virus, Raji cells were infected with serial 10-fold dilutions of virus. After 24 hours, cells were treated with 50 ng/ml TPA and 3 mM sodium butyrate, and the number of GFP-expressing Raji cells was counted 24–48 hours later by fluorescence microscopy.

### EBV infection of B cells

B cells were centrifuged and resuspended in media containing wildtype, Zp -141 mutant, or revertant B95.8 virus (produced by 293 cell lines and titered on Raji cells) for a total volume of 500uL and an MOI of 0.1 or 0.25 (primary B cells) or 1 (BJAB, Akata and Mutu cells). Cells and virus were incubated for 1–3 h with occasional stirring, then media was increased to 4 mL for overnight incubation. The next day cells were spun down and resuspended in fresh media, except for human peripheral blood CD19+ B cells, which were not centrifuged. Peripheral blood CD19+ B cells and Akata cells infected with EBV were harvested at day 3 post-infection, and BJAB cells at 6 days after infection, and extracts containing equal amounts of protein used for immunoblots. Mutu cells were selected with 300 ug/mL hygromycin B starting at day five post-infection to create stable cell lines; Mutu lines were under selection for at least two months before other experiments were performed.

### GFP FACS analysis

To determine the number of EBV-infected CD19+ B cells, and the mean fluorescence intensity, a portion of cells was analyzed with a LSRII flow cytometer (BD Biosciences) three days post-infection. Data analysis was performed using FlowJo software.

### siRNA experiments

EBV-infected Mutu cells were nucleofected with 120 pmol control siRNA (Santa Cruz sc-37007) or NFATc1 siRNA (29412) using Amaxa program N-16 in Buffer V. Ionomycin or DMSO control was added after 48 hours. Cells were harvested 72 hours post-nucleofection and immunoblots performed using equal amounts of protein.

### Chromatin Immunoprecipitation assay (ChIP)

EBV-infected Mutu cells treated with ionomycin for 3hrs were harvested and fixed with 1% formaldehyde in PBS for 8 minutes at room temperature followed by addition of glycine to 125mM for 5 minutes at room temperature to quench the reaction. Fixed cells were pelleted, washed once with PBS and twice with Cell Lysis/Wash Buffer (150mM NaCl, 50mM Tris pH 7.4, 5mM EDTA pH 8.0, 0.5% NP-40, 1% Triton X-100). Pellets were resuspended in ChIP Lysis Buffer (50mM Tris pH 8.0, 10mM EDTA pH 8.0, 1% SDS) and chromatin was sheared by sonication using a QSonica Q700 sonicator (3 rounds of 10 cycles of 30sec on/30sec off at 95% amplitude in an ice water bath). Debris was cleared by centrifugation at 11,500 x g for 10 min at 4°C. Supernatant was then diluted 1:5 in ChIP Dilution Buffer (16.7mM Tris pH 8.0, 167mM NaCl, 1.2mM EDTA, 1.1% Triton X-100, 0.01% SDS) and chromatin from approximately one million cells was incubated with 3ug of rabbit anti-NFATc1 antibody (Bethyl Laboratories A303-508A) or rabbit IgG control antibody (Millipore 12–370) overnight at 4°C. Chromatin/antibody complexes were isolated with Magna ChIP Protein A+G magnetic beads (Millipore 16–663) and subsequently washed with Low Salt Buffer (20mM Tris pH 8.0, 150mM NaCl, 2mM EDTA, 1% Triton X-100, 0.1% SDS), High Salt Buffer (20mM Tris pH 8.0, 0.5M NaCl, 1% Triton X-100, 0.1% SDS), LiCl Buffer (10mM Tris pH 8.0, 0.25M LiCl, 1mM EDTA pH 8.0, 1% NP-40, 1% DOC), and TE (10mM Tris pH 8.0, 1mM EDTA pH 8.0). Crosslinks were reversed and DNA was isolated with an IBI Gel/PCR DNA fragment extraction kit (IB47030; IBI) and quantitated by qPCR using iTaq Universal SYBR Green Supermix (172–5124; Bio-Rad) and primers to the Z promoter (FWD 5′-GCCATGCATATTTCAACTGGGCTG-3′ and REV 5′-TGCCTGTGGCTCATGCATAGTTTC-3′) and analyzed using an ABI 7900HT real-time PCR system with SDS2.4 software (Applied Biosystems).

### EBV transformation assay

Transformation titration assays were performed by infecting human peripheral blood CD19+ B cells (10,000 cells/well in a 96-well microtiter plate) with 0.25 infectious GFP Raji Units/cell (10 replicates per virus) and culturing with RPMI complete medium. Wells with clearly growing LCLs (lymphoblastoid cell lines) were scored microscopically after at least 3 weeks of culture.

### Statistical analysis of frequency of Zp-V3 in samples from healthy individuals versus Burkitt lymphomas and gastric carcinomas

A portion of the Zp sequences analyzed in this paper (many of which were also previously analyzed for Zp type) were derived from publicly available sequences of EBV genomes deposited in Genbank. Z promoter sequences that were not previously analyzed for the type of Zp variant present are aligned in S6 Table. Zp promoters were considered to have Zp-V3 if they contained the Zp-V3 specific nucleotide located at position -141.

In addition to Genbank EBV sequences, the TCGA database was interrogated for EBV-positive gastric cancers (stomach adenocarcinoma or STAD) by using the Genomic Data Commons Application program interface (GDC-API) to perform BAM slicing on harmonized TCGA data. Reads mapping to "chrEBV" were sliced and Samtools was used to quantify the number of reads with MAPQ of 20 or greater. Tumor EBV read count was a bimodal distribution with 27 tumors having 2,888–53,779 reads and 379 having 0–217 reads and one tumor with 534 reads. All tumors with >500 reads were treated as EBV-positive.

Zp variant calling was performed on all 28 EBV-positive samples obtained from the TCGA database using any informative reads that could be obtained from RNAseq and whole exome sequencing (WXS) data for each tumor. The GDC-API was used to perform BAM slicing on GDC harmonized TCGA data from chrEBV in the region 91006–47. Zp-P or Zp-V3 calls were made based on the following position: nt91006 "-100" Zp-P = A Zp-v3 = C; nt91012 "-106" Zp-P = T Zp-v3 = C; nt91047 "-141" Zp-P = T Zp-V3 = C. For 5 samples for which neither RNAseq nor WXS provided informative coverage of the Z promoter, whole genome sequencing data was obtained from the GDC legacy archive. Reads mapping to NC_007605 in the BAM files were manually reviewed in IGV 2.3.82 and Zp-P or Zp-V3 calls were made as above. To estimate the background prevalence of each Zp genotype in the TCGA database, blood and normal tissue WXS (whole exome sequencing) datasets from TCGA were interrogated using the GDC-API to perform BAM slicing for reads mapping to "chrEBV." Samtools was used to identify samples containing reads from chrEBV: 91006–91047 with MAPQ of 20 or greater. These BAM files were then manually viewed in IGV 2.3.82 and Zp-P or Zp-V3 calls were made based as for tumor tissues. Additional criteria included requiring base quality of 10 or greater at the call position and the mate read had to also map to chrEBV.

Available RNA-seq data from endemic Burkitt tumors were also downloaded from sequence read archive (SRA) PRJNA292327 [58]. Fastq files were then aligned to the type 1 and 2 EBV genomes (AJ507799.2 and NC_009334.1) using BWA’s backtrack algorithm with default settings.

Zp alignment for TCGA samples was performed as follows. BAM slices from GDC for each sample were evaluated in IGV v2.3.82. Consensus quality scores were calculated by summing individual Phred scores when reads had different mates. Bases with consensus quality scores less than 20 or without read coverage were represented with a (-). Bases with a consensus quality score of 20 or more were represented with an uppercase letter.

### BZLF1 promoter analysis of breast-milk derived EBV-transformed LCLs

LCLs were transformed by infectious EBV particles present in the breast milk of Kenyan women that had malaria during pregnancy living in a rural region of Kisumu County, as previously described [17]. DNA was isolated from these LCLs and the EBV Zp amplified by PCR using the following primers: Zp+34 primer 5’-GCAAAGATAGCAAAGGTGGC and Zp-561 primer 5’-GAACCGGTCGGATCCCTAAC. Sequencing of the Zp promoter was performed using the Zp +34 primer.

### Statistical analyses

The program Mstat, Version 6.1, was used for all statistical analyses (N. Drinkwater, McArdle Laboratory for Cancer Research, School of Medicine and Public Health, University of Wisconsin) and is available for downloading (http://www.mcardle.wisc.edu/mstat).

### Ethics statement

The primary human peripheral CD19+ B cells from healthy donors used in this study are considered exempt by the University of Wisconsin-Madison Institutional Review Board (IRB). These cells were purchased from Stem Cell Technologies (#70033), which obtained written donor consent using IRB-approved protocols.

The anonymized EBV breast milk samples used for this study have been previously documented [17]. The original study received approval from the Kenya Medical Research Institute (KEMRI), the University of Colorado COMIRB, and Upstate Medical University (where R. Rochford was at initiation of study) Review Boards. Written informed consent was obtained from all study participants before any sample collection.

## Supporting information

S1 Table The Burkitt lymphoma samples used to calculate the frequency of Zp-V3 containing type 1 EBV genomes in Burkitt lymphomas occurring in African or South American countries are shown, along with the sample type, geographic location, EBV type, Z promoter variant, PubMed ID (when available), and Genbank accession number. The one T1/T2 recombinant genome was considered T1 for this analysis.
(DOCX)

S2 Table Non-malignant samples (spontaneous LCLs from healthy patients in Kenya, or PBMCs from infectious mononucleosis (IM) patients in Argentina) used as geographic controls for the BLs in Tables 1 and 2 are shown. The one recombinant T1/T2 sample was considered T1, and the IM sample containing both Zp-P and Z-V3 was not included in the analysis.
(DOCX)

S3 Table Samples listed were used to calculate the frequency of Zp-V3 containing T1 EBV genomes in gastric carcinomas occurring in Asian versus non-Asian patients. The source, geographic location, EBV type, Z promoter variant, Race, Genbank accession or TCGA ID Numbers (when available) and PubMed ID (when available) are shown. The one T1/T2 recombinant genome was considered T1 for this analysis.
(DOCX)

S4 Table Non-malignant samples (spontaneous LCLs from healthy or IM patients in the USA, Australia, or Italy, PBMCs from infectious mononucleosis (IM) patients in Massachusetts, USA, and contaminating EBV genomes in the TCGA data base) used as known or presumed non-Asian controls for the gastric carcinomas occurring in non-Asian patients in Table 5 are shown. (DOCX)

S5 Table Non-malignant samples that were used as known (or presumed) Asian controls for the gastric carcinomas occurring in Asian patients in Table 4 included contaminating EBV genomes in the TCGA database from Asian individuals as shown above. In addition, other controls (all presumed to be Asian) included in the analysis were EBV genomes isolated from saliva of 21 healthy individuals in China (22), or 15 PBMCs from infectious mononucleosis (IM) patients in China (22), or PBMCs from 38 healthy children in China (71). Samples were considered to be the Zp-V3 variant if they had the Zp-V3–141 variant nucleotide.
(DOCX)

S6 Table The BZLF1 promoter sequences that have not been previously annotated as Zp-P versus Zp-V3 are shown. The 3 bp nucleotide differences in the two promoter forms are highlighted in yellow (Zp-P) and green (Zp-V3). Samples were considered to be the Zp-V3 variant if they had the Zp-V3–141 variant nucleotide, or contained both the -100 and -106 Zp-V3 variant nucleotides with an un-sequenced -141 nucleotide (TCGA samples).
(DOCX)
