# Supplementary material for: A cancer-associated Epstein-Barr virus BZLF1 promoter variant enhances lytic infection
Source: PLoS Pathog. 2018 Jul 27;14(7):e1007179. doi: 10.1371/journal.ppat.1007179 (PMC6082571; doi:10.1371/journal.ppat.1007179)
Supplement: S1 Table — The one T1/T2 recombinant genome was considered T1 for this analysis. (DOCX) [file ppat.1007179.s001.docx]

**Supplemental Table 1**.

**Burkitt lymphomas**

| **Sample** | **Geographic Origin** | **Sample Type** | **EBV Type** | **Zp-P/V3** | **PubMed ID** | **Accession Number** |
| --- | --- | --- | --- | --- | --- | --- |
| GK BL60 | N.Africa | BL cell line | 1 | P | 28515295 | MG298836 |
| GK LY65 | Uganda | BL cell line | 1 | P | 28515295 | MG298841 |
| GK BL18 | N.Africa | BL cell line | 1 | P | 28515295 | MG298832 |
| GK BL72 | N.Africa | BL cell line | 1 | P | 28515295 | MG298838 |
| GK LY47 | Uganda | BL cell line | 1 | P | 28515295 | MG298840 |
| JW BL17A | Africa | BL cell line | 1 | P | 28515295 | MG298904 |
| JW BL121B | Africa | BL cell line | 1 | P | 28515295 | MG298903 |
| Daudi | Africa | BL cell line | 1 | P | 28515295 | LN827545 |
| BL37 | Africa | BL cell line | 1 | P | 28515295 | LN827526 |
| Raji | Nigeria | BL cell line | 1 | P | 28515295 | KF717093 |
| Mutu | Kenya | BL cell line | 1 | P | 28515295 | KC207814 |
| GK Akuba | Nairobi | BL cell line | 1 | V3 | 28515295 | MG298830 |
| GK BL42 | N.Africa | BL cell line | 1 | V3 | 28515295 | MG298834 |
| GK BL44 | N.Africa | BL cell line | 1 | V3 | 28515295 | MG298835 |
| GK BL36 | N.Africa | BL cell line | 1 | V3 | 28515295 | LN827557 |
| JW BL43B | Africa | BL cell line | 1 | V3 | 28515295 | MG298905 |
| Makau | Africa | BL cell line | 1 | V3 | 28515295 | LN827551 |
| Mak-1 | Africa | BL cell line | 1 | V3 | 28515295 | LN824203 |
| BL36 | Africa | BL cell line | Recombinant | V3 | 28515295 | LN827557 |
| eBL23 | Uganda | BL tumor | 1 | P | 26468873 | SRX1137161 |
| eBL30 | Uganda | BL tumor | 1 | P | 26468873 | SRX1137255 |
| eBL40 | Uganda | BL tumor | 1 | P | 26468873 | SRX1137194 |
| eBL69 | Uganda | BL tumor | 1 | P | 26468873 | SRX1137209 |
| eBL80 | Uganda | BL tumor | 1 | P | 26468873 | SRX1137210 |
| eBL22 | Uganda | BL tumor | 1 | V3 | 26468873 | SRX1137158 |
| H002213 | Ghana | BL tumor | 1 | P | 28515295 | KP968264 |
| HU11393 | Ghana | BL tumor | 1 | P | 28515295 | KP968261 |
| HO3753A | Ghana | BL tumor | 1 | P | 28515295 | KR063342 |
| H058015C | Ghana | BL tumor | 1 | V3 | 28515295 | KP968263 |
| H018436D | Ghana | BL tumor | 1 | V3 | 28515295 | KP968262 |
| MP | Brazil | BL tumor | 1 | P | 26593963 | KP968258 |
| CCH | Brazil | BL tumor | 1 | P | 26593963 | KP968257 |
| FNR | Brazil | BL tumor | 1 | P | 26593963 | KR063345 |
| CV-ARG | Argentina | BL tumor | 1 | P | 26593963 | KR063343 |
| SG | Argentina | BL tumor | 1 | P | 26593963 | KT001103 |
| **Sample** | **Geographic Origin** | **Sample Type** | **EBV Type** | **Zp-P/V3** | **PubMed ID** | **Accession Number** |
| SCL | Brazil | BL tumor | 1 | V3 | 26593963 | KP968259 |
| RPF | Brazil | BL tumor | 1 | V3 | 26593963 | KR063344 |
| T3 | Argentina | BL tumor | 1 | V3 | 19774688 | FJ756536 |
| GK LY91 | Uganda | BL cell line | 2 | V3 | 28515295 | MG298918 |
| GK BL16 | Africa | BL cell line | 2 | V3 | 28515295 | MG298831 |
| Jijoye | Africa | BL cell line | 2 | V3 | 28515295 | LN827800 |
| Cheptages | Africa | BL cell line | 2 | V3 | 28515295 | LN827556 |
| AG876 | W. Africa | BL cell line | 2 | V3 | 28515295 | NC009334 |
| eBL35 | Uganda | BL tumor | 2 | V3 | 26468873 | SRX1137191 |
| eBL81 | Uganda | BL tumor | 2 | V3 | 26468873 | SRX1137212 |
