# Supplementary material for: A cancer-associated Epstein-Barr virus BZLF1 promoter variant enhances lytic infection
Source: PLoS Pathog. 2018 Jul 27;14(7):e1007179. doi: 10.1371/journal.ppat.1007179 (PMC6082571; doi:10.1371/journal.ppat.1007179)
Supplement: S2 Table — The one recombinant T1/T2 sample was considered T1, and the IM sample containing both Zp-P and Z-V3 was not included in the analysis. (DOCX) [file ppat.1007179.s002.docx]

**Supplemental Table 2**.

**Control Samples for Burkitt lymphoma analysis**

| **Sample** | **Geographic Origin** | **Sample Type** | **EBV Type** | **Zp-P/V3** | **PubMed ID** | **Accession Number** |
| --- | --- | --- | --- | --- | --- | --- |
| **Spontaneous LCLs** | | | | | | |
| sLCL-1.02 | Kenya | sLCL | 1 | P | 28515295 | LN827588 |
| sLCL-BL1.03 | Kenya | sLCL | 1 | P | 28515295 | LN827582 |
| sLCL-1.04 | Kenya | sLCL | 1 | P | 28515295 | LN827585 |
| sLCL-1.05 | Kenya | sLCL | 1 | P | 28515295 | LN827581 |
| sLCL-1.06 | Kenya | sLCL | 1 | P | 28515295 | LN827566 |
| sLCL-1.07 | Kenya | sLCL | 1 | P | 28515295 | LN827565 |
| sLCL-1.08 | Kenya | sLCL | 1 | P | 28515295 | LN827552 |
| sLCL-1.09 | Kenya | sLCL | 1 | P | 28515295 | LN827574 |
| sLCL-1.10 | Kenya | sLCL | 1 | P | 28515295 | LN827573 |
| sLCL-1.11 | Kenya | sLCL | 1 | P | 28515295 | LN827550 |
| sLCL-1.12 | Kenya | sLCL | 1 | P | 28515295 | LN824205 |
| sLCL-1.13 | Kenya | sLCL | 1 | P | 28515295 | LN827579 |
| sLCL-1.17 | Kenya | sLCL | 1 | P | 28515295 | LN827577 |
| sLCL-1.19 | Kenya | sLCL | 1 | P | 28515295 | LN827562 |
| sLCL-BL1.20 | Kenya | sLCL | 1 | P | 28515295 | LN827571 |
| sLCL-1.24 | Kenya | sLCL | 1 | P | 28515295 | LN827568 |
| sLCL-1.18 | Kenya | sLCL | Recombinant | V3 | 28515295 | LN827563 |
| sLCL-2.14 | Kenya | sLCL | 2 | V3 | 28515295 | LN827560 |
| sLCL-2.15 | Kenya | sLCL | 2 | V3 | 28515295 | LN827591 |
| sLCL-2.16 | Kenya | sLCL | 2 | V3 | 28515295 | LN827580 |
| sLCL-2.21 | Kenya | sLCL | 2 | V3 | 28515295 | LN827587 |
| sLCL-2.22 | Kenya | sLCL | 2 | V3 | 28515295 | LN831023 |
| **IM Samples** | | | | | | |
| P1 | Argentina | PBMC | 1 | P | 19774688 | FJ756491 |
| P2 | Argentina | PBMC | 1 | P | 19774688 | FJ756497 |
| P3 | Argentina | PBMC | 1 | P | 19774688 | FJ756499 |
| P4 | Argentina | PBMC | 1 | P | 19774688 | FJ756501 |
| P5 | Argentina | PBMC | 1 | P | 19774688 | FJ756507 |
| P6 | Argentina | PBMC | 1 | P | 19774688 | FJ756513 |
| P8 | Argentina | PBMC | 1 | P | 19774688 | FJ756523 |
| P9 | Argentina | PBMC | 1 | P/V3 | 19774688 | FJ756529 |
| P10 | Argentina | PBMC | 1 | P | 19774688 | FJ756531 |
| P7 | Argentina | PBMC | 2 | V3 | 19774688 | FJ756517 |
