# Supplementary material for: A cancer-associated Epstein-Barr virus BZLF1 promoter variant enhances lytic infection
Source: PLoS Pathog. 2018 Jul 27;14(7):e1007179. doi: 10.1371/journal.ppat.1007179 (PMC6082571; doi:10.1371/journal.ppat.1007179)
Supplement: S3 Table — The source, geographic location, EBV type, Z promoter variant, Race, Genbank accession or TCGA ID Numbers (when available) and PubMed ID (when available) are shown. The one T1/T2 recombinant genome was considered T1 for this analysis. (DOCX) [file ppat.1007179.s003.docx]

**Supplemental Table 3**.

**Gastric Carcinomas**

| **Sample** | **Geographic Origin** | **Sample**  **Type** | **EBV Type** | **Zp-P/V3** | **Race** | **TCGA ID/**  **Accession #** | **PubMed ID** |
| --- | --- | --- | --- | --- | --- | --- | --- |
| GC1 | China | GC | 1 | P | Asian | KT273949 | 26716899 |
| GC5 | China | GC | 1 | P | Asian | KT273945 | 26716899 |
| GC2 | China | GC | 1 | V3 | Asian | KT273943 | 26716899 |
| GC3 | China | GC | 1 | V3 | Asian | KT254013 | 26716899 |
| GC4 | China | GC | 1 | V3 | Asian | KT273944 | 26716899 |
| GC6 | China | GC | 1 | V3 | Asian | KT273946 | 26716899 |
| GC7 | China | GC | 1 | V3 | Asian | KT273947 | 26716899 |
| GC8 | China | GC | 1 | V3 | Asian | KT273948 | 26716899 |
| GC9 | China | GC | 1 | V3 | Asian | KT273949 | 26716899 |
| YCCEL1 | S. Korea | GC | 1 | P | Asian | AP015016 | 26889033 |
| SNU719 | S. Korea | GC | 1 | V3 | Asian | KP735248 | 26459384 |
| GC-Var1 | China | GC | 1 | V3 | Asian | MG021314 | 29093097 |
| GC-Var2 | China | GC | 1 | P | Asian | MG021305.1 | 29093097 |
| GC-Var3 | China | GC | 1 | V3 | Asian | MG021315 | 29093097 |
| GC-Var4 | USA | GC | 1 | V3 | Asian | MG021317 | 29093097 |
| GC-Var5 | USA | GC | 1 | P | Caucasian | MG021308 | 29093097 |
| GC-Var6 | USA | GC | 1 | V3 | Asian | MG021307 | 29093097 |
| GC-Var7 | USA | GC | 1 | V3 | Asian | MG021312 | 29093097 |
| GC-Var8 | USA | GC | 1 | P | Asian | MG021316 | 29093097 |
| GC-Var9 | USA | GC | 1 | P | Caucasian | MG021310 | 29093097 |
| GC-Var10 | USA | GC | 1 | P | Caucasian | MG021311 | 29093097 |
| GC-Var11 | USA | GC | 1 | P | Caucasian | MG021309 | 29093097 |
| GC-Var12 | USA | GC | 1 | V3 | Asian | MG021313 | 29093097 |
| 1 | Poland | GC | 1 | P | Caucasian | TCGA-D7-A4YX |  |
| 2 | Poland | GC | 1 | P | Caucasian | TCGA-D7-8573 |  |
| 3 | Poland | GC | 1 | P | Caucasian | TCGA-D7-8570 |  |
| 4 | Russia | GC | 1 | P | Caucasian | TCGA-BR-6455 |  |
| 5 | Russia | GC | 1 | P | Caucasian | TCGA-B7-5818 |  |
| 6 | Russia | GC | 1 | P | Caucasian | TCGA-BR-7958 |  |
| 7 | Ukraine | GC | 1 | P | Caucasian | TCGA-BR-8381 |  |
| 8 | Ukraine | GC | 1 | P | Caucasian | TCGA-BR-6707 |  |
| 9 | USA | GC | 1 | P | Caucasian | TCGA-FP-7998 |  |
| 10 | Vietnam | GC | 1 | P | Asian | TCGA-BR-A4J4 |  |
| 11 | Vietnam | GC | 1 | P | Asian | TCGA-BR-8686 |  |
| 12 |  | GC | 1 | P | Not Reported | TCGA-VQ-A8PF |  |
| **Sample** | **Geographic Origin** | **Sample Type** | **EBV Type** | **Zp-P/V3** | **Race** | **TCGA ID** | **PubMed ID** |
| 13 |  | GC | 1 | P | Caucasian | TCGA-B7-A5TK |  |
| 14 | Germany | GC | 1 | V3 | Not Reported | TCGA-CG-5722 |  |
| 15 | Poland | GC | 1 | V3 | Caucasian | TCGA-D7-5577 |  |
| 16 | Russia | GC | 1 | V3 | Caucasian | TCGA-BR-4253 |  |
| 17 | Ukraine | GC | 1 | V3 | Caucasian | TCGA-BR-8366 |  |
| 18 | Vietnam | GC | 1 | V3 | Asian | TCGA-CD-5801 |  |
| 19 | S. Korea | GC | 2 | V3 | Asian | TCGA-HU-8608 |  |
