# Supplementary material for: A cancer-associated Epstein-Barr virus BZLF1 promoter variant enhances lytic infection
Source: PLoS Pathog. 2018 Jul 27;14(7):e1007179. doi: 10.1371/journal.ppat.1007179 (PMC6082571; doi:10.1371/journal.ppat.1007179)
Supplement: S4 Table — (DOCX) [file ppat.1007179.s004.docx]

**Supplemental Table 4**.

**Control samples for Caucasian Gastric carcinoma analysis**

| **USA Infectious Mononucleosis Sequences** | | | | | | | |
| --- | --- | --- | --- | --- | --- | --- | --- |
| **Sample** | **Geographic Origin** | **Sample Type** | **EBV Type** | **Zp-P/V3** | **Race** | **Accession Number** | **PubMed ID** |
| E1583_BCv1 | USA | PBMCs | 1 | P | Not Reported | MF547453 | 29093087 |
| E1587_BCv1 | USA | PBMCs | 1 | P | Not Reported | MF547457 | 29093087 |
| E1536_BCv1 | USA | PBMCs | 1 | P | Not Reported | MF547461 | 29093087 |
| E1563_BCv1 | USA | PBMCs | 1 | P | Not Reported | MF547463 | 29093087 |
| E1548_BCv1 | USA | PBMCs | 1 | P | Not Reported | MF547466 | 29093087 |
| E1590_BCv1 | USA | PBMCs | 1 | P | Not Reported | MF547473 | 29093087 |
| E1492_BCv1 | USA | PBMCs | 1 | P | Not Reported | MF547477 | 29093087 |
| E1503_BCv1 | USA | PBMCs | 1 | P | Not Reported | MF547481 | 29093087 |
| E1578_BCv1 | USA | PBMCs | 1 | P | Not Reported | MF547485 | 29093087 |
| E1577_BCv1 | USA | PBMCs | 1 | P | Not Reported | MF547489 | 29093087 |
|  | | | | | | | |
| **sLCLs and immortalized B-cell lines from Australian and USA infectious mononucleosis (IM) patients** | | | | | | | |
| **Sample** | **Geographic Origin** | **Sample Type** | **EBV Type** | **Zp-P/V3** | **Race** | **Accession Number** | **PubMed ID** |
| sLCL-IM1.02 | Australia | sLCL, IM | 1 | P | Not Reported | LN827596 | 28515295 |
| sLCL-IM1.05 | Australia | sLCL, IM | 1 | P | Not Reported | LN827590 | 28515295 |
| sLCL-IM1.09 | Australia | sLCL, IM | 1 | P | Not Reported | LN827567 | 28515295 |
| sLCL-IM1.16 | Australia | sLCL, IM | 1 | P | Not Reported | LN827799 | 28515295 |
| sLCL-IM1.17 | Australia | sLCL, IM | 1 | P | Not Reported | LN827583 | 28515295 |
| K4123 | USA | sLCL, IM | 1 | P | Not Reported | KC440851 | 28515295 |
| B95.8 | USA | Marmoset transformed B cell line, IM patient | 1 | P | Not Reported | NC_007605 | 28515295 |
| X50-7 | USA | LCL | 1 | P | Not Reported | LN827555 | 28515295 |
|  | | | | | | | |
| **sLCL from healthy Italian donors** | | | | | | | |
| **Sample** | **Geographic Origin** | **Sample Type** | **EBV Type** | **Zp-P/V3** | **Race** | **Accession Number** | **PubMed ID** |
| CAR | Italy | sLCL | 1 | P | Not Reported |  | 27635051 |
| NM | Italy | sLCL | 1 | P | Not Reported |  | 27635051 |
| MC | Italy | sLCL | 1 | P | Not Reported |  | 27635051 |
| GIOVS | Italy | sLCL | 1 | P | Not Reported |  | 27635051 |
| GR | Italy | sLCL | 1 | P | Not Reported |  | 27635051 |
| PT | Italy | sLCL | 1 | P | Not Reported |  | 27635051 |
| BR | Italy | sLCL | 1 | P | Not Reported |  | 27635051 |
| **Sample** | **Geographic Origin** | **Sample Type** | **EBV Type** | **Zp-P/V3** | **Race** | **Accession Number** | **PubMed ID** |
| LUL | Italy | sLCL | 1 | P | Not Reported |  | 27635051 |
| MST | Italy | sLCL | 1 | P | Not Reported |  | 27635051 |

| **Contaminating EBV genomes in samples from patients in TCGA Database** | | | | | | |
| --- | --- | --- | --- | --- | --- | --- |
| **Sample** | **Geographic Origin** | **Sample Type** | **EBV Type** | **Zp-P/V3** | **Race** | **TCGA ID** |
| 1 | Not Reported | Ovary | 1 | P | Caucasian | TCGA-13-0757 |
| 2 | Not Reported | Ovary | 1 | P | Caucasian | TCGA-13-0916 |
| 3 | Not Reported | Ovary | 1 | P | Caucasian | TCGA-13-0923 |
| 4 | Not Reported | Lung | 1 | P | Caucasian | TCGA-44-2665 |
| 5 | Not Reported | Ovary | 1 | P | Caucasian | TCGA-61-2095 |
| 6 | Not Reported | Lung | 1 | P | Caucasian | TCGA-67-3773 |
| 7 | Not Reported | GBM | 1 | P | Caucasian | TCGA-76-4929 |
| 8 | Not Reported | Prostate | 1 | P | Caucasian | TCGA-EJ-5508 |
| 9 | Not Reported | Esophagus | 1 | P | Caucasian | TCGA-L5-A88S |
