# Supplementary material for: A cancer-associated Epstein-Barr virus BZLF1 promoter variant enhances lytic infection
Source: PLoS Pathog. 2018 Jul 27;14(7):e1007179. doi: 10.1371/journal.ppat.1007179 (PMC6082571; doi:10.1371/journal.ppat.1007179)
Supplement: S5 Table — In addition, other controls (all presumed to be Asian) included in the analysis were EBV genomes isolated from saliva of 21 healthy individuals in China (22), or 15 PBMCs from infectious mononucleosis (IM) patients in China (22), or PBMCs from 38 healthy children in China (71). Samples were considered to be the Zp-V3 variant if they had the Zp-V3–141 variant nucleotide. (DOCX) [file ppat.1007179.s005.docx]

**Supplemental Table 5**.

**Control samples for Asian Gastric carcinoma analysis**

| **Contaminating EBV genomes in samples from patients in TCGA Database** | | | | | | | | | | | | |  |
| --- | --- | --- | --- | --- | --- | --- | --- | --- | --- | --- | --- | --- | --- |
| **Sample** | **Geographic Origin** | | **Sample Type** | | **EBV Type** | | **Zp-P/V3** | | **Race** | | **TCGA ID** | |  |
| 1 | Not Reported | | Lung | | 1 | | P | | Asian | | TCGA-55-1595 | |  |
| 2 | Not Reported | | Stomach | | 1 | | P | | Asian | | TCGA-HU-8602 | |  |
| 3 | Vietnam | | Esophagus | | 1 | | P | | Asian | | TCGA-LN-A49W | |  |
|  | |  | |  | |  | |  | |  | |  | |
