# Supplementary material for: A cancer-associated Epstein-Barr virus BZLF1 promoter variant enhances lytic infection
Source: PLoS Pathog. 2018 Jul 27;14(7):e1007179. doi: 10.1371/journal.ppat.1007179 (PMC6082571; doi:10.1371/journal.ppat.1007179)
Supplement: S6 Table — The 3 bp nucleotide differences in the two promoter forms are highlighted in yellow (Zp-P) and green (Zp-V3). Samples were considered to be the Zp-V3 variant if they had the Zp-V3–141 variant nucleotide, or contained both the -100 and -106 Zp-V3 variant nucleotides with an un-sequenced -141 nucleotide (TCGA samples). (DOCX) [file ppat.1007179.s006.docx]

**Supplemental Table 6**.

**Zp sequences of malignant and non-malignant samples**

**Type 1 Burkitt Lymphomas**

-100 -106 -141

* * *

eBL23 1 GGTGTGTCTATGAGGTACATTAGCAATGCCTGTGGCTCATGCATAGTTTCTAAAAGAGG 59 zp-P

eBL30 1 GGTGTGTCTATGAGGTACATTAGCAATGnnTGTGGCTCATGCATAGTTTCTAAAAGAGG 59 zp-P

eBL40 1 GGTGTGTCTATGAGGTACATTAGCAATGCCTGTGGCTCATGCATAGTTTCTAAA----- 59 zp-P

eBL69 1 GGTGTGTCTATGAGGTACATTAGCAATGCCTGTGGCTCATGCATAGTTTCTAAAAGAGG 59 zp-P

eBL80 1 ------------------------------TGTGGCTCATGCATAGTTTCTAAAAGAGG 59 zp-P

eBL22 1 GGTGTGTCTCTGAGGCACATTAGCAATGCCTGTGGCTCATGCATAGTTTCCAAAAGAGG 59 zp-v3

H002213 1 ggtgtgtctatgaggtacattagcaatgcctgtggctcatgcatagtttctaaaagagg 59 zp-P

HU11393 1 ggtgtgtctatgaggtacattagcaatgcctgtggctcatgcatagtttctaaaagagg 59 zp-P

HO3753A 1 ggtgtgtctatgaggtacattagcaatgcctgtggctcatgcatagtttctaaaagagg 59 zp-P

H058015C 1 ggtgtgtctatgaggtacattagcaatgcctgtggctcatgcatagtttccaaaagagg 59 zp-V3

H018436D 1 ggtgtgtctatgaggtacattagcaatgcctgtggctcatgcatagtttccaaaagagg 59 zp-V3

MP 1 ggtgtgtctatgaggtacattagcaatgcctgtggctcatgcatagtttctaaaagagg 59 zp-P

CCH 1 ggtgtgtctatgaggtacattagcaatgcctgtggctcatgcatagtttctaaaagagg 59 zp-P

FNR 1 ggtgtgtctatgaggtacattagcaatgcctgtggctcatgcatagtttctaaaagagg 59 zp-P

CV-ARG 1 ggtgtgtctatgaggtacattagcaatgcctgtggctcatgcatagtttctaaaagagg 59 zp-P

SG 1 ggtgtgtctatgaggtacattagcaatgcctgtggctcatgcatagtttctaaaagagg 59 zp-P

SCL 1 ggtgtgtctctgaggcacattagcaatgcctgtggctcatgcatagtttccaaaagagg 59 zp-V3

RPF 1 ggtgtgtctctgaggcacattagcaatgcctgtggctcatgcatagtttccaaaagagg 59 zp-V3

eBL35 1 GGTGTGTCTCTGAGGCACATTAGCAATGCCTGT-------------------------- 59 zp-v3

eBL81 1 GGTGTGTCTCTGAGGCACATTAGCAATGCCTGTGGCTCATGCATAGTTTCCAAAAGAGG 59 zp-v3

**Gastric Carcinomas**

-100 -106 -141

* * *

GC1 1 GGTGTGTCTATGAGGTACATTAGCAATGCCTGTGGCTCATGCATAGTTTCCAAAAGAGG 59 zp-V3

GC2 1 GGTGTGTCTCTGAGGCACATTAGCAATGCCTGTGGCTCATGCATAGTTTCCAAAAGAGG 59 zp-V3

GC3 1 ggtgtgtctctgaggcacattagcaatgcctgtggctcatgcatagtttccaaaagagg 59 zp-V3

GC4 1 ggtgtgtctctgaggcacattagcaatgcctgtggctcatgcatagtttccaaaagagg 59 zp-V3

GC5 1 ggtgtgtctatgaggtacattagcaatgcctgtggctcatgcatagtttctaaaagagg 59 zp-P

GC6 1 ggtgtgtctctgaggcacattagcaatgcctgtggctcatgcatagtttccaaaagagg 59 zp-V3

GC7 1 ggtgtgtctctgaggcacattagcaatgcctgtggctcatgcatagtttccaaaagagg 59 zp-V3

-100 -106 -141

* * *

GC8 1 ggtgtgtctctgaggcacattagcaatgcctgtggctcatgcatagtttccaaaagagg 59 zp-V3

GC9 1 ggtgtgtctatgaggtacattagcaatgcctgtggctcatgcatagtttccaaaagagg 59 zp-V3

YCCEL1 1 ggtgtgtctatgaggtacattagcaatgcctgtggctcatgcatagtttctaaaagagg 59 zp-P

SNU719 1 ggtgtgtctctgaggcacattagcaatgcctgtggctcatgcatagtttccaaaagagg 59 zp-V3

GC-Var1 1 ggtgtgtctctgaggcacattagcaatgcctgtggctcatgcatagtttccaaaagagg 59 zp-V3

GC-Var1 1 ggtgtgtctatgaggtacattagcaatgcctgtggctcatgcatagtttctaaaagagg 59 zp-P

GC-Var3 1 ggtgtgtctctgaggcacattagcaatgcctgtggctcatgcatagtttccaaaagagg 59 zp-V3

GC-Var4 1 ggtgtgtctctgaggcacattagcaatgcctgtggctcatgcatagtttccaaaagagg 59 zp-V3

GC-Var5 1 ggtgtgtctatgaggtacattagcaatgcctgtggctcatgcatagtttctaaaagagg 59 zp-P

GC-Var6 1 ggtgtgtctctgaggcacattagcaatgcctgtggctcatgcatagtttccaaaagagg 59 zp-V3

GC-Var7 1 ggtgtgtctctgaggcacattagcaatgcctgtggctcatgcatagtttccaaaagagg 59 zp-V3

GC-Var8 1 ggtgtgtctatgaggtacattagcaatgcctgtggctcatgcatagtttctaaaagagg 59 zp-P

GC-Var9 1 ggtgtgtctatgaggtacattagcaatgcctgtggctcatgcatagtttctaaaagagg 59 zp-P

GC-Var10 1 ggtgtgtctatgaggtacattagcaatgcctgtggctcatgcatagtttctaaaagagg 59 zp-P

GC-Var11 1 ggtgtgtctatgaggtacattagcaatgcctgtggctcatgcatagtttctaaaagagg 59 zp-P

GC-Var12 1 ggtgtgtctctgaggcacattagcaatgcctgtggctcatgcatagtttccaaaagagg 59 zp-V3

TCGA-D7-A4YX 1 GGTGTGTCTATGAGGTACATTAGCAATGCCTGTGGCTCATGCATAGTTTCTAAAAGAGG 59 zp-P

TCGA-D7-8573 1 GGtGtGtCTATGAgGTACatTAGCAATgcCTGTGG--CATgCATagTTTCTAAAAGAGG 59 zp-P

TCGA-D7-8570 1 GGTGTGTCTATGAGGTACATTAGCAATGCCTGTGGCTCATGCATAGTTTCTAAAAGAGG 59 zp-P

TCGA-BR-6455 1 GGTG-GtCTAtGaGGTACAttaGCAatGcCtGTGGCTCATGCATAGtTTCTAAAAGAGG 59 zp-P

TCGA-B7-5818 1 GGTGTGTCTATGAGGTACATTAGCAATGCCTGTGGCTCATGCATAGTTTCTAAAAGAGG 59 zp-P

TCGA-BR-7958 1 GGTGTGTCTATGAGGTACATTAGCAATGCCTGTGGCTCATGCATAGTTTCTAAAAGAGG 59 zp-P

TCGA-BR-8381 1 GGTGTGTCTATGAGGTACATTAGCAATGCCTGTGGCTCATGCATAGTTTCTAAAAGAGG 59 zp-P

TCGA-BR-6707 1 GGTGTGTCTATGAGGTACATTAGCAATGCCTGTGGCTCATGCATAGTTTCTAAAAGAGG 59 zp-P

TCGA-FP-7998 1 GGTGTGTCTATGAGGTACATTAGCAATGCCTGTGGCTCATGCATAGTTTCTAAAAGAGG 59 zp-P

TCGA-BR-A4J4 1 GGTGTGTCTATGAGGTACATTAGCAATGCCTGTGGCTCATGCATAGTTTCTAAAAGAGG 59 zp-P

TCGA-BR-8686 1 GGTGTGTCTATGAGGtaCATTAGCAATGCCTGTGgCTCATGCATAGTTTCTaAAAGAGG 59 zp-P

TCGA-VQ-A8PF 1 GGTGTGTCTATGAGGTACATTAGCAATGCCTGTGGCTCATGCATAGTTTCTAAAAGAGG 59 zp-P

TCGA-B7-A5TK 1 GGTGTGTCTCTGAGGCACATTAGCAATGCCTGTGGCTCATGCATAGTTTCCAAAAGAGG 59 zp-V3

TCGA-CG-5722 1 GGTGTGTCTCTGAGGCACATTAGCAATGCCTGTGGCTCATGCATAGTTTCCAAAAGAGG 59 zp-V3

TCGA-D7-5577 1 GGTGTGTCTCTGAGGCACATTAGCAATGCCTGTGGCTCATGCATAGTTTCCAAAAGAGG 59 zp-V3

TCGA-BR-4253 1 GGTGTGTCTCTGAGGCACATTAGCAATGCCTGTGGCTCATGCATAGTTTCCAAAAGAGG 59 zp-V3

TCGA-BR-8366 1 --TGTGTCTCTGAGGCACATTAGCAATGCCTGTGGCTCATGCATAGTTTCCaAAAGAGG 59 zp-V3

TCGA-CD-5801 1 GGTGTGTCTCTGAGGCACATtAGCa---------------------------------- 59 zp-V3

-100 -106 -141

* * *

TCGA-HU-8608 1 ggTgTgtCtCTGagGC-CattagCaaTgCCTgTGGCtCaTGcatagtttCCaaaaGaGG 59 zp-V3

**USA infectious mononucleosis patients**

-100 -106 -141

* * *

E1583_BCv1 1 ggtgtgtctatgaggtacattagcaatgcctgtggctcatgcatagtttctaaaagagg 59 zp-P

E1587_BCv1 1 ggtgtgtctatgaggtacattagcaatgcctgtggctcatgcatagtttctaaaagagg 59 zp-P

E1536_BCv1 1 ggtgtgtctatgaggtacattagcaatgcctgtggctcatgcatagtttctaaaagagg 59 zp-P

E1563_BCv1 1 ggtgtgtctatgaggtacattagcaatgcctgtggctcatgcatagtttctaaaagagg 59 zp-P

E1548_BCv1 1 ggtgtgtctatgaggtacattagcaatgcctgtggctcatgcatagtttctaaaagagg 59 zp-P

E1590_BCv1 1 ggtgtgtctatgaggtacattagcaatgcctgtggctcatgcatagtttctaaaagagg 59 zp-P

E1492_BCv1 1 ggtgtgtctatgaggtacattagcaatgcctgtggctcatgcatagtttctaaaagagg 59 zp-P

E1503_BCv1 1 ggtgtgtctatgaggtacattagcaatgcctgtggctcatgcatagtttctaaaagagg 59 zp-P

E1578_BCv1 1 ggtgtgtctatgaggtacattagcaatgcctgtggctcatgcatagtttctaaaagagg 59 zp-P

E1577_BCv1 1 ggtgtgtctatgaggtacattagcaatgcctgtggctcatgcatagtttctaaaagagg 59 zp-P

**Italy spontaneous LCLs from healthy donors**

-100 -106 -141

* * *

CAR 1 GGTGTGTCTATGAGGTACATTAGCAATGCCTGTGGCTCATGCATAGTTTCTAAAAGAGG 59 zp-P

NM 1 GGTGTGTCTATGAGGTACATTAGCAATGCCTGTGGCTCATGCATAGTTTCTAAAAGAGG 59 zp-P

MC 1 GGTGTGTCTATGAGGTACATTAGCAATGCCTGTGGCTCATGCATAGTTTCTAAAAGAGG 59 zp-P

GR 1 GGTGTGTCTATGAGGTACATTAGCAATGCCTGTGGCTCATGCATAGTTTCTAAAAGAGG 59 zp-P

PT 1 GGTGTGTCTATGAGGTACATTAGCAATGCCTGTGGCTCATGCATAGTTTCTAAAAGAGG 59 zp-P

BR 1 GGTGTGTCTATGAGGTACATTAGCAATGCCTGTGGCTCATGCATAGTTTCTAAAAGAGG 59 zp-P

LUL 1 GGTGTGTCTATGAGGTACATTAGCAATGCCTGTGGCTCATGCATAGTTTCTAAAAGAGG 59 zp-P

GIOVS 1 GGTGTGTCTATGAGGTACATTAGCAATGCCTGTGGCTCATGCATAGTTTCTAAAAGAGG 59 zp-P

MST 1 GGTGTGTCTATGAGGTACATTAGCAATGCCTGTGGCTCATGCATAGTTTCTAAAAGAGG 59 zp-P

**Contaminating EBV genomes in samples in TCGA**

-100 -106 -141

* * *

TCGA-13-0757 1 GGTGTGTCTATGAGGTACATTAGCAATGCCTGTGGCTCATGCATAGTTTCTAAAAGAGG 59 zp-P

-100 -106 -141

* * *

TCGA-13-0916 1 GGTGTGTCTATGAGGTACATTAGCAAT-------------------------------- 59 Zp-P

TCGA-13-0923 1 -----------GAGGTACATTAGCAATGCCTGTGGCTCATGCATAG-TTCTAAAAGAGG 59 Zp-P

TCGA-44-2665 1 GGTGTGTCTATGAGGTACATTAGCAATGCCTGTGGCTCATG------------------ 59 Zp-P

TCGA-61-2095 1 GGTGTGTCTATGAGGTACATTAGCAATGCCTGTGGCTCATGCATAGTTTCTAAAAGAGG 59 Zp-P

TCGA-67-3773 1 GGTGTGTCTATGAGGTACATTAGCAATGCCTGTGGCTCATGCATAGTTTCTAAAAGAGG 59 Zp-P

TCGA-76-4929 1 GGTGTGTCTATGAGGTACAT---------CTGTGGCTCATGCATAGTTTCTAAAAGAGG 59 Zp-P

TCGA-EJ-5508 1 GGTGTGTCTATGAGGTACATTAGCAATGCCTGTGGCTCATGCATAGT------------ 59 Zp-P

TCGA-L5-A88S 1 -------------------------------------CATGCATAGTTTCTAAAAGAGG 59 Zp-P

TCGA-55-1595 1 GGTGTGTCTATGAGGTACATTAGCAATGCCTGTGGCTCATGCATAGTTTCTAAAAGAGG 59 Zp-P

TCGA-HU-8602 1 -–TGTGTCTATGAGGTACATTAGCAATGCCTGTGGCTCATGCATAGTTTCTAAAAGAGG 59 Zp-P

TCGA-LN-A49W 1 GGTGTGTCTATGAGGTACATTAG------------------------------------ 59 Zp-P
